# Landscape of X chromosome inactivation across human tissues

**DOI:** 10.1038/nature24265

**Published:** 2017-10-11

**Authors:** Taru Tukiainen, Alexandra-Chloé Villani, Angela Yen, Manuel A. Rivas, Jamie L. Marshall, Rahul Satija, Matt Aguirre, Laura Gauthier, Mark Fleharty, Andrew Kirby, Beryl B. Cummings, Stephane E. Castel, Konrad J. Karczewski, François Aguet, Andrea Byrnes, GTEx Consortium, Tuuli Lappalainen, Aviv Regev, Kristin G. Ardlie, Nir Hacohen, Daniel G. MacArthur

**Affiliations:** 1 Analytic and Translational Genetics Unit, Massachusetts General Hospital, Boston, MA 02114, USA; 2 Broad Institute of MIT and Harvard, Cambridge, MA 02142, USA; 3 Center for Immunology and Inflammatory Diseases, Massachusetts General Hospital, Charlestown, MA 02129, USA; 4 Computer Science and Artificial Intelligence Laboratory, Massachusetts Institute of Technology, Cambridge, MA 02139, USA; 5 Department of Biomedical Data Science, Stanford University, Stanford, CA 94305, USA; 6 New York Genome Center, New York, NY 10013, USA; 7 Center for Genomics and Systems Biology, Department of Biology, New York University, New York, NY 10003, USA; 8 Department of Systems Biology, Columbia University, New York, NY 10032, USA; 9 Department of Biology, Massachusetts Institute of Technology, Cambridge, MA 02139, USA

X chromosome inactivation (XCI) silences the transcription from one of the two X chromosomes in mammalian female cells to balance expression dosage between XX females and XY males. XCI is, however, characteristically incomplete in humans: up to one third of X-chromosomal genes are expressed from both the active and inactive X chromosomes (Xa and Xi, respectively) in female cells, with the degree of “escape” from inactivation varying between genes and individuals^1,2^ (Fig. 1). However, the extent to which XCI is shared between cells and tissues remains poorly characterized^3,4^, as does the degree to which incomplete XCI manifests as detectable sex differences in gene expression^5^ and phenotypic traits^6^. Here we report a systematic survey of XCI integrating over 5,500 transcriptomes from 449 individuals spanning 29 tissues from GTEx (V6 release), and 940 single-cell transcriptomes, combined with genomic sequence data (Fig. 1). We show that XCI at 683 X-chromosomal genes is generally uniform across human tissues, but identify examples of heterogeneity between tissues, individuals, and cells. We show that incomplete XCI affects at least 23% of X-chromosomal genes, identify seven new escape genes supported by multiple lines of evidence, and demonstrate that escape from XCI results in sex biases in gene expression, establishing incomplete XCI as a likely mechanism introducing phenotypic diversity^6,7^. Overall, this updated catalogue of XCI across human tissues informs our understanding of the extent and impact of the incompleteness in the maintenance of XCI.

Mammalian female tissues consist of two mixed cell populations, each with either the maternally or paternally inherited X chromosome marked for inactivation. To overcome this heterogeneity, assessments of human XCI have often been confined to the use of artificial cell systems^1^, or samples presenting with skewed XCI^1,2^, i.e. preferential inactivation of one of the two X chromosomes, which is common in clonal cell lines but rare in karyotypically normal, primary human tissues^8^ (Supplementary Note, Extended Data Fig. 1). Others have used bias in DNA methylation^3,4,9^ or in gene expression^5,10^ between males and females as a proxy for XCI status. Surveys of XCI are powerful in engineered model organisms, e.g. mouse models with completely skewed XCI^11^, but the degree to which these discoveries are generalizable to human XCI remains unclear given marked differences in XCI initiation and extent of escape across species^7^. Here, we describe a systematic survey of the landscape of human XCI using three complementary RNA sequencing (RNA-seq)-based approaches (Fig. 1) that together allow an assessment of XCI from individual cells to population across a diverse range of human tissues.

Given the limited accessibility of most human tissues, particularly in large sample sizes, no global investigation of the impact of incomplete XCI on X-chromosomal expression has been conducted in data sets spanning multiple tissue types. We used the Genotype Tissue Expression (GTEx) project^12^ data set (V6 release), which includes high-coverage RNA-seq data from diverse human tissues, to investigate male-female differences in the expression of 681 X-chromosomal protein-coding and long non-coding RNA (lncRNA) genes in 29 adult tissues (Extended Data Table 1), hypothesizing that escape from XCI should typically result in higher female expression of these genes. Previous work^5,10,13^ has indicated that some escape genes show female bias in expression, but our analysis benefits from a larger set of profiled tissues and individuals, as well as the high sensitivity of RNA-seq.

To confirm that male-female expression differences reflect incomplete XCI, we assessed the enrichment of sex-biased expression in known XCI categories using 561 genes with previously assigned XCI status, defined as escape (N=82), variable escape (N=89) or inactive (N=390)(Fig. 1, Supplementary Table 1). Sex-biased expression is enriched in escape genes compared to both inactive (two-sided paired Wilcoxon P=3.73×10^-9^) and variable escape genes (P=3.73×10^-9^) (Fig. 2b, Extended Data Fig. 2), with 74% of escape genes showing significant (false discovery rate (FDR) q-value < 0.01) male-female differences in at least one tissue (Fig. 2a, Extended Data Fig. 3-4, Supplementary Table 2). In line with two active X-chromosomal copies in females, escape genes in the non-pseudoautosomal, i.e. the X-specific, region (nonPAR) predominantly show female-biased expression across tissues (52 out of 67 assessed genes, binomial P=6.46×10^-6^). However, genes in the pseudoautosomal region PAR1, are expressed more highly in males (14/15 genes, binomial P=9.77×10^-6^) (Fig. 2a), suggesting that combined Xa and Xi expression in females fails to reach the expression arising from X and Y chromosomes in males (discussed below).

Sex bias of escape genes is often shared across tissues; these genes show a higher number of tissues with sex-biased expression than genes in other XCI categories (Fig. 2a, Extended Data Fig. 2c), a result not driven by differences in the breadth of expression of escape and inactive genes (Extended Data Fig. 2e).Also, the direction of sex bias across tissues is consistent (Fig. 2a,c, Extended Data Fig. 2b). Together these observations point toward global and tight control of XCI, potentially arising from early lockdown of the epigenetic marks regulating XCI. Previous reports have identified several epigenetic signatures associated with XCI escape in human and mouse^14^; in agreement with these discoveries we find that escape genes are enriched in chromatin states related to active transcription (Fig. 2e).

While sex bias on the X chromosome is broadly specific to escape genes, some genes show unexpected patterns. Eight genes with some previous evidence for inactivation show >90% concordance in effect direction and significant sex bias (Fig. 2d, Supplementary Table 3), e.g. *CHM*, that replicates in single-cell RNA-seq (scRNA-seq; see below), suggesting that variable escape can also have considerable population-level impact. One gene without an assigned XCI status shows a similar sex bias pattern to escape genes; *RP11-706O15.3* (Fig. 2d) resides between escape and variable escape genes *PRKX* and *NLGN4X*, consistent with known clustering of escape genes^1,2^. Some escape genes show more heterogeneous sex bias, e.g., *ACE2* (Fig. 2a, Supplementary Discussion). Many of such genes lie in the evolutionarily older region of the chromosome^15^, in Xq, where escape genes also show higher tissue-specificity and lower expression levels (Extended Data Fig. 5), characteristics linked with higher protein evolutionary rates^16,17^.

While sex bias serves as a proxy for XCI status, it provides only an indirect measure of XCI. We identified a GTEx female donor with an unusual degree of skewing of XCI (Fig. 3a), the same copy of chrX being silenced in ∼100% of cells across all tissues, yet without any X-chromosomal abnormality detected by whole-genome sequencing (WGS) (Supplementary Note, Extended Data Fig. 6), providing an opportunity to leverage allele-specific expression (ASE) across 16 tissues to investigate XCI. This approach is analogous to previous surveys in mouse^11^ or in human cell lines with skewed XCI^2^, but extends the assessment to larger number of tissues and avoids biases arising from genetic heterogeneity between tissue samples.

Analysis of the X-chromosomal allelic counts (Supplementary Tables 4-6) from this GTEx donor highlights the incompleteness and consistency of XCI across tissues (Fig. 3b). Approximately 23% of the 186 X-chromosomal genes assessed show expression from both alleles, indicative of incomplete XCI, matching previous estimates of the extent of escape^1,2^. For 43% of the genes expressed biallelically in this sample, Xi expression is of similar magnitude between tissues, thus supporting the observation of general global and tight control of XCI. However, suggesting some tissue-dependence in XCI, the rest of biallelically expressed genes show variability in Xi expression, including a gene subset (5.8% of all genes) that appear biallelic in only one of the multiple tissues assayed. While tissue-specific escape is common in mouse^11^, limited evidence exists for such a pattern in human tissues beyond neurons^3,4,9^. In our data, among the genes with the strongest evidence for tissue-specific escape is *KAL1* (Fig. 3f, Supplementary Table 6), the causal gene for X-linked Kallmann syndrome; here *KAL1* shows biallelic expression exclusively in lung (Fig. 3f), in line with the strong female bias detected specifically in lung expression in the previous analysis (Fig. 2a), suggesting that tissue differences in escape can directly translate to tissue-specific sex biases in gene expression. Altogether, the predictions of XCI status in this sample align with previous assignments (Supplementary Table 7, e.g. *TSR2*, *XIST* and *ZBED1,* Fig. 3c-f), but suggest five new incompletely inactivated genes (Fig. 3g-k, Supplementary Table 5), three of which act in a tissue-specific manner. For instance,*CLIC2*, in previous studies called either subject^2^ to or variably escaping^1^ from XCI, shows considerable Xi expression only in skin tissue. Such specific patterns illustrate the need to assay multiple tissue types to fully uncover the diversity in XCI.

The emergence of scRNA-seq methods^18^ presents an opportunity to directly assess XCI without the complication of cellular heterogeneity in bulk tissue samples (Fig. 1), as demonstrated recently in mouse studies^19-22^, and in human fibroblasts^23^ and preimplantation development^24^. To directly profile XCI in human samples, we examined scRNA-seq data in combination with deep genotype sequences from 940 immune-related cells from four females: 198 cells from LCLs sampled from three females of African (Yoruba) ancestry, and 742 blood dendritic cells from a female of Asian ancestry^25^ (Fig. 1, Extended Data Table 2). We utilized ASE to distinguish the expression coming from each of the two X-chromosomal haplotypes in a given cell (Supplementary Table 4). As the inference of allele-specific phenomena in single cells is complicated by widespread monoallelic expression^20,26-28^, besides searching for X-chromosomal sites with biallelic expression (Extended Data Fig. 7), we leveraged genotype phase information to detect sites where the expressed allele was discordant with the active X chromosome in that cell.

Only 129 (78%) out of the 165 assayed genes (41-98 per sample)were fully inactivated in these data while the rest showed incomplete XCI in one or more samples (Fig. 4a-b, Supplementary Tables 8-9), largely consistent with previous assignments of XCI status to these genes (Fig. 4a, Supplementary Table 10). For instance, single cell data reveal consistent expression from both X-chromosomal alleles for eleven genes in PAR1, in line with their known escape from XCI (e.g. *ZBED1*, Fig. 4c), and replicate the known expression of *XIST* exclusively from Xi (Fig. 4d).

We next assessed whether our approach could extend the spectrum of escape from XCI. For seven genes previously reported as inactivated the data from single cells pointed to incomplete XCI (Fig. 4e-k, Supplementary Table 11), including *FHL1* (Fig. 4e), highlighted as a candidate escape gene also in the GTEx ASE analysis, and *ATP6AP2* (Fig. 4h), which displays predominantly female-biased expression across GTEx tissues. Both of these genes demonstrate significant Xi expression in only a subset of the scRNA-seq samples, a pattern consistent with variable escape^1,2^. Between-individual variability exists not only in the presence but also in the degree of expression from Xi (e.g. *MSL3*, Fig. 4l). Highlighting the capacity of scRNA-seq to provide information beyond bulk RNA-seq, we identify examples where Xi expression varies considerably between the two X-chromosomal haplotypes within an individual (e.g. *ASMTL*; Supplementary Table 12), suggesting *cis*-acting variation as one of the determinants for the level of Xi expression^3^. As a further layer of heterogeneity in Xi expression, we find a unique pattern at *TIMP1*, where the level of Xi expression across cells is not significant, but exclusive to a subset of cells that express the gene biallelically (Extended Data Fig. 7), pointing to cell-to-cell variability in escape.

Leveraging the ASE estimates from the scRNA-seq and GTEx analyses to infer the magnitude of the incompleteness of XCI,we find that expression from Xi at escape genes rarely reaches levels equal to Xa. Xi expression remains on average at 33% of Xa expression, yet with wide variability along the chromosome (Supplementary Discussion, Extended Data Fig. 8a), as demonstrated previously in specific tissue types^1,2^. Balanced expression dosage between males and females in PAR1 requires full escape from XCI, yet Xi expression remains below Xa expression also in this region (mean Xi to Xa ratio ∼0.80), pointing to partial spreading of XCI beyond nonPAR. For further support for the consistent male bias in PAR1 expression (Fig. 2a) being due to the incompleteness of escape, we observe no systematic up- or downregulation of Y chromosome expression in PAR1 (Extended Data Fig. 8b, Supplementary Discussion). As another consequence of the partial Xi expression, several of the X-Y homologous genes in nonPAR^29^ become male-biased when expression from the Y chromosome counterpart is accounted for (Extended Data Fig. 8c).

By combining diverse types and analyses of high-throughput RNA-seq data, we have systematically assessed the incompleteness and heterogeneity in XCI across 29 human tissues (Supplementary Table 13). We establish that scRNA-seq is suitable for surveys of human XCI and present the first steps towards understanding the cellular-level variability in the maintenance of XCI. Our phasing-based approach allows for the full use of low-coverage scRNA-seq, yet as any single individual and cell type is informative for restricted number of genes, larger data sets with more diverse cell types and conditions are required to fully profile XCI. We have thus utilized the multi-tissue GTEx data set to explore XCI in a larger number of X-chromosomal genes and to assess the tissue-heterogeneity and impacts of XCI on gene expression differences between the sexes.

These analyses show that incomplete XCI is largely shared between individuals and tissues, and extend previous surveys by pinpointing several examples of variability in the degree of XCI escape between cells, chromosomes, and tissues. In addition, our data demonstrate that escape from XCI results in sex-biased expression in at least 60 genes, potentially contributing to sex differences in health and disease (Supplementary Discussion). As a whole, these results highlight the between-female and male-female diversity introduced by incomplete XCI, the biological implications of which remain to be fully explored.

## Methods

### GTEx data

The GTEx project^12^ collected tissue samples from 554 postmortem donors (187 females, 357 males; age range 20-70), produced RNA sequencing from 8,555 tissue samples and generated genotyping data for up to 449 donors (GTEx Analysis V6 release). More details of methods can be found in Aguet et al. (Aguet et al., *co-submitted, Nature*). All GTEx data, including RNA, genome and exome sequencing data, used in the analyses described are available through dbGaP under accession phs000424.v6.p1, unless otherwise stated. Summary data and details on data production and processing are also available on the GTEx Portal (http://gtexportal.org).

### Single-cell samples

For the human dendritic cells samples profiled, the healthy donor (ID: 24A) was recruited from the Boston-based PhenoGenetic project, a resource of healthy subjects that are re-contactable by genotype^30^. The donor was a female Asian individual from China, of 25 years of age at the time of blood collection. She was a non-smoker, had normal BMI (height: 168.7cm; weight: 56.45kg; BMI: 19.8), and normal blood pressure (108/74). The donor had no family history of cancer, allergies, inflammatory disease, autoimmune disease, chronic metabolic disorders or infectious disorders. She provided written informed consent for the genetic research studies and molecular testing, as previously reported^25^.

Daughters of three parent-child Yoruba trios from Ibadan, Nigeria, (i.e. YRI trios) collected as part of the International HapMap Project, were chosen for single-cell profiling both to maximize heterozygosity and due to availability of parental genotypes allowing for phasing. DNA and LCLs were ordered from the NHGRI Sample Repository for Human Genetic Research (Coriell Institute for Medical Research): LCLs from B-Lymphocyte for the three daughters (catalogue numbers: GM19240, GM19199, GM18518) and DNA extracted from LCLs for all members of the three trios (catalogue numbers: DNA: NA19240, NA19238, NA19239, NA19199, NA19197, NA19198, NA18518, NA18519, NA18520). These YRI samples are referred to by their family IDs: Y014, Y035 and Y117.

### Clinical muscle samples

To assess whether PAR1 genes are equally expressed from X and Y chromosomes, a combination of skeletal muscle RNA sequencing and trio genotyping from eight male patients with muscular dystrophy, sequenced as part of an unrelated study, was used. Patient cases with available muscle biopsies were referred from clinicians starting April 2013 through June 2016. All patients submitted for RNA-sequencing had previously available trio whole exome sequencing with one sample having additional trio whole genome sequencing. Muscle biopsies were shipped frozen from clinical centers via liquid nitrogen dry shipper and, where possible, frozen muscle was sectioned on a cryostat and stained with H&E to assess muscle quality as well as the presence of overt freeze-thaw artifact.

### Genotyping

The GTEx V6 release includes WGS data for 148 donors, including GTEX-UPIC. WGS libraries were sequenced on the Illumina HiSeqX or Illumina HiSeq2000. WGS data was processed through a Picard-based pipeline, using base quality score recalibration and local realignment at known indels. BWA-MEM aligner was used for mapping reads to the human genome build 37 (hg19). SNPs and indels were jointly called across all 148 samples and additional reference genomes using GATK's HaplotypeCaller version 3.1. Default filters were applied to SNP and indel calls using the GATK's Variant Quality Score Recalibration (VQSR) approach. An additional hard filter InbreedingCoeff <= -0.3 was applied to remove sites that VQSR failed to filter.

WGS for one of the clinical muscle samples was performed on 500 ng to 1.5 ug of genomic DNA using a PCR-Free protocol that substantially increases the uniformity of genome coverage. These libraries were sequenced on the Illumina HiSeq × 10 with 151 bp paired-end reads and a target mean coverage of >307times;, and processed similarly as above.

The Y117 trio (sample IDs NA19240 (daughter), NA19238 (mother), and NA19239 (father)) was whole-genome-sequenced as part of the 1000 Genomes project as described previously^31^. The VCF file containing the WGS-based genotypes for SNPs (YRI.trio.2010_09.genotypes.vcf.gz) was downloaded from the project's FTP site. The genotype coordinates (in human genome build 36) in the original VCF were converted to hg19 using the liftover script (liftOverVCF.pl) and chain files provided as part of the GATK package.

WES was performed using Illumina's capture Exome (ICE) technology (Y035, Y014, 24A) or Agilent SureSelect Human All Exon Kit v2 exome capture (clinical muscle samples) with a mean target coverage of >80×. WES data was aligned with BWA, processed with Picard, and SNPs and indels were called jointly with other samples using GATK HaplotypeCaller package version 3.1 (24A, clinical muscle samples) or version 3.4 (Y035, Y014). Default filters were applied to SNP and indel calls using the GATK's Variant Quality Score Recalibration (VQSR) approach. A modified version of the Ensembl Variant Effect Predictor was used for variant annotation for all WES and WGS data. For trio WES or WGS data the genotypes of the proband were phased using the PhaseByTransmission tool of the GATK toolkit.

### Single cell data preparation and sequencing

For profiling of healthy DCs, peripheral blood mononuclear cells (PBMCs) were first isolated from fresh blood within 2hrs of collection, using Ficoll-Paque density gradient centrifugation as previously described^32^. Single-cell suspensions were stained per manufacturer recommendations with an antibody panel designed to enrich for all known blood DC population for single cell sorting and single cell RNA-sequencing (scRNA-seq) profiling^25^. A total of 24 single cells from four loosely gated populations were sorted per 96-well plate, with each well containing 10ul of lysis buffer. A total of eight plates were analysed by single-cell RNA-sequencing.

All LCL cell lines were cultured according to Coriell's recommendation (medium: RPMI 1640, 2mM L-glutamine, 15% fetal bovine serum (all three from ThermoFisher Scientific)) in T25 tissue culture flask with 10-20 ml medium at 37°C under 5% carbon dioxide. Cells were split upon reaching cell density of approximately 300,000-400,000 viable cells/ml. All three lymphoblast cultures were split once prior to proceeding with single cell sorting. Cells were washed with 1× PBS, pellet resuspended and stained with DAPI (Biolegend) for viability according to manufacturer's recommendation.

All single live cells (for both DCs and LCL cell lines) were sorted in 96-well full-skirted eppendorf plate chilled to 4°C, pre-prepared with 10μl TCL buffer (Qiagen) supplemented with 1% beta-mercaptoethanol (lysis buffer) using BD FACS Fusion instrument. Single-cell lysates were sealed, vortexed, spun down at 300g at 4°C for 1 minute, immediately placed on dry ice and transferred for storage at -80°C.

The Smart-Seq2 protocol was performed on single sorted cells as described^33,34^, with some modifications as described in Villani et al.^25^ (Supplementary Methods). A total of 768 single DCs isolated from healthy Asian female individual, along with 96 single cells from GM19240, 48 single cells from GM19199, and 48 single cells from GM18518 were profiled. Briefly, single-cell lysates were thawed on ice purified, and reverse-transcribed using Maxima H Minus Reverse Transcriptase. PCR was performed with KAPA HiFi HotStart ReadyMix [KAPA Biosystems] and purified with Agencourt AMPureXP SPRI beads (Beckman-Coulter). The concentration of amplified cDNA was measured on the Synergy H1 Hybrid Microplate Reader (BioTek) using High-Sensitivity Qubit reagent (Life Technologies), and the size distribution of select wells was checked on a High-Sensitivity Bioanalyzer Chip (Agilent). Expected quantification was around 0.5-2 ng/μL with size distribution sharply peaking around 2kb.

Library preparation was carried out using the Nextera XT DNA Sample Kit (Illumina) with custom indexing adapters, allowing up to 384 libraries to be simultaneously generated in a 384-well PCR plate (note that DCs were processed in 384-well plate while LCL were processed in 96-well plate format). The concentration of the final pooled libraries was measured using the High-Sensitivity DNA Qubit (Life Technologies), and the size distribution measured on a High-Sensitivity Bioanalyzer Chip (Agilent). Expected concentration of the pooled libraries was 10-30 ng/μL with size distribution of 300-700bp. For the DCs, we created pools of 384 cells, while 96 LCL samples were pooled at the time. We sequenced one library pool per lane as paired-end 25 base reads on a HiSeq2500 (Illumina). Barcodes used for indexing are listed in the Supplementary Methods.

### RNA-seq in GTEx

RNA sequencing was performed using a non-strand-specific RNA-seq protocol with poly-A selection of RNA using the Illumina TruSeq protocol with sequence coverage goal of 50M 76 bp paired-end reads as described in detail previously^12^. The RNA-seq data, except for GTEX-UPIC, was aligned with Tophat version v1.4.1 to the UCSC human genome release version hg19 using the Gencode v19 annotations as the transcriptome reference. Gene level read counts and RPKMs were derived using the RNA-SeQC tool^35^ using the Gencode v19 transcriptome annotation. The transcript model was collapsed into gene model as described previously^12^. Read count and RPKM quantification include only uniquely mapped and properly paired reads contained within exon boundaries.

### RNA-seq alignment to personalized genomes

For the four single-cell samples and for GTEX-UPIC RNA-seq data was processed using a modification of the AlleleSeq pipeline^36,37^ to minimize reference allele bias in alignment. A diploid personal reference genome for each of the samples was generated with the vcf2diploid tool^36^ including all heterozygous biallelic single nucleotide variants identified in WES or WGS either together with (YRI samples) or without (GTEX-UPIC, 24A) maternal and paternal genotype information. The RNA-seq reads were then aligned to both parental references using STAR^38^ version 2.4.1a in a per-sample 2-pass mode (GTEX-UPIC and YRI samples) or version 2.3.0e (24A) using hg19 as the reference. The alignments were combined by comparing the quality of alignment between the two references: for reads aligning uniquely to both references the alignment with the higher alignment score was chosen and reads aligning uniquely to only one reference were kept as such.

### RNA-seq of clinical muscle samples

Patient RNA samples derived from primary muscle were sequenced using the GTEx sequencing protocol^12^ with sequence coverage of 50M or 100M 76 bp paired-end reads. RNA-seq reads were aligned using STAR^38^ 2-Pass version v.2.4.2a using hg19 as the reference. Junctions were filtered after first pass alignment to exclude junctions with less than 5 uniquely mapped reads supporting the event and junctions found on the mitochondrial genome. The value for unique mapping quality was assigned to 60 and duplicate reads were marked with Picard MarkDuplicates (v.1.1099).

### Catalogue of X-inactivation status

In order to compare results from the ASE and GTEx analyses with previous observations on genic XCI status we collated findings from two earlier studies^1,2^ that represent systematic expression-based surveys into XCI. Each study catalogues hundreds of X-linked genes and together the data span two tissue types.

Carrel and Willard^1^ surveyed in total 624 X-chromosomal transcripts expressed in primary fibroblasts in nine cell hybrids each containing a different human Xi. In order to find the gene corresponding to each transcript, the primer sequences designed to test the expression of the transcripts in the original study were aligned to reference databases based on Gencode v19 transcriptome and hg19 using an in-house software (unpublished) (Supplementary Methods). In total 553 transcripts primer pairs were successfully matched to X-chromosomal Gencode v19 reference mapping together to 470 unique chrX genes (Supplementary Methods). These 470 genes were split into three XCI status categories (escape, variable, inactive) based on the level of Xi expression (i.e. the number of cell lines expressing the gene from Xi) resulting in 75 escape, 51 variable escape and 344 inactive genes.

Cotton et al^2^ surveyed XCI using allelic imbalance in clonal or near-clonal female LCL and fibroblast cell lines and provided XCI statuses for 508 genes (68 escape, 146 variable escape, 294 subject genes). The data was mapped to Gencode v19 using the reported gene names and their known aliases (Supplementary Methods), resulting in a list of XCI statuses for 506 X-chromosomal genes.

The results were combined by retaining the XCI status in the original study where possible (i.e. same status in both studies or gene unique to one study) and for genes where the reported XCI statuses were in conflict the following rules were applied: 1) A gene was considered “escape” if it was called escape in one study and variable in the other, 2) “variable escape” if classified as escape and inactive, and 3) “inactive” if classified as inactive in one study and variable escape in the other. The final combined list of XCI statuses consisted of 631 X-chromosomal genes including 99 escape, 101 variable escape and 431 inactive genes.

### Analysis of sex-biased expression

Differential expression analyses were conducted to identify genes that are expressed at significantly different levels between male and female samples using 29 GTEx V6 tissues with RNA-seq and genotype data available from more than 70 individuals after excluding samples flagged in QC and sex-specific, outlier (i.e. breast tissue) and highly correlated tissues^13^. Only autosomal and X-chromosomal protein-coding or lncRNA genes in Gencode v19 were included, and further all lowly-expressed genes were removed. (Supplementary Methods and Extended Data Table 1).

Differential expression analysis between male and female samples was conducted following the voom-limma pipeline^39-41^ available as an R package through Bioconductor (https://bioconductor.org/packages/release/bioc/html/limma.html) using the gene-level read counts as input. The analyses were adjusted for age, three principal components inferred from genotype data using EIGENSTRAT^42^, sample ischemic time, surrogate variables^43,44^ built using the sva R package^45^, and the cause of death classified into five categories based on the 4-point Hardy scale (Supplementary Methods).

To control the false discovery rate (FDR), the qvalue R package was used to obtain q-values applying the adjustment separately for the differential expression results from each tissue. The null hypothesis was rejected for tests with q-values below 0.01.

### XY homolog analysis

A list of Y-chromosomal genes with functional counterparts in the X chromosome, i.e. X-Y gene pairs, was obtained from Bellott et al^29^, which lists 19 ancestral Y chromosome genes that have been retained in the human Y chromosome. After excluding two of the genes (*MXRA5Y* and *OFD1Y*), which were annotated as pseudogenes by Bellot et al and further four genes (*SRY*, *RBMY*, *TSPY*, and *HSFY*) that according to Bellot et al have clearly diverged in function from their X-chromosomal homologs, the remaining 13 Y-chromosomal genes were matched with their X chromosome counterparts using gene pair annotations given in Bellot et al or by searching for known paralogs of the Y-chromosomal genes. To test for completeness of dosage compensation at the X-Y homologous genes, the sex bias analysis in GTEx data was repeated replacing the expression of the X-chromosomal counterpart with the combined expression of the X and Y homologs.

### Chromatin state analysis

To study the relationship between chromatin states and XCI, we used chromatin state calls from the Roadmap Epigenomics Consortium^46^. Specifically, we used the chromatin state annotations from the core 15-state model, publicly available at http://egg2.wustl.edu/roadmap/web_portal/chr_state_learning.html#core_15state. We followed our previously published method^47^ to calculate the covariate-corrected percentage of each gene body assigned to each chromatin state. After pre-processing, we filtered down to the 399 inactive and 86 escape genes on the X chromosome, and down to 38 female epigenomes.

To compare the chromatin state profiles of the escape and inactive genes in female samples, we used the one-sided Wilcoxon rank sum test. Specifically, for each chromatin state, we averaged the chromatin state coverage across the 38 female samples for each gene, and compared that average chromatin state coverage for all 86 escape genes to the average chromatin state coverage for all 399 inactive genes. We performed both one-sided tests, to test for enrichment in escape genes, as well as enrichment in inactive genes.

Next, we performed simulations to account for possible chromatin state biases, such as the fact that the escape and inactive genes are all from the X chromosome. Specifically, we generated 10,000 randomized simulations where we randomly shuffled the “escape” or “inactive” labels on the combined set of 485 genes, while retaining the sizes of each gene set. For each of these simulated “escape” and “inactive” gene sets, we calculated both one-sided Wilcoxon rank sum test p-values as described above, and then, we calculated a permutation “p-value” for the real gene sets based on these 10,000 random simulations (Supplementary Methods). Finally, we used Bonferroni multiple hypothesis correction for our significance thresholds to correct for our 30 tests, one for each of 15 chromatin states, and both possible test directions.

### Allele-specific expression

For ASE analysis the allele counts for biallelic heterozygous variants were retrieved from RNA-seq data using GATK ASEReadCounter (v.3.6)^37^. Heterozygous variants that passed VQSR filtering were first extracted for each sample from WES or WGS VCFs using GATK SelectVariants. The analysis was restricted to biallelic SNPs due to known issues in mapping bias in RNA-seq against indels^37^. Sample-specific VCFs and RNA-seq BAMs were inputted to ASEReadCounter requiring minimum base quality of 13 in the RNA-seq data (scRNA-seq samples, GTEX-UPIC) or requiring coverage in the RNA-seq data of each variant to be at least 10 reads, with a minimum base quality of 10 and counting only reads with unique mapping quality (MQ = 60) (clinical muscle samples).

For downstream processing of the scRNA-seq and GTEX-UPIC ASE data, we applied further filters to the data to focus on exonic variation only and to conservatively remove potentially spurious sites (Supplementary Methods), e.g. sites with non-unique mappability were removed, and further, after an initial analysis of the ASE data, subjected 22 of the X-chromosomal ASE sites to manual investigation. For GTEX-UPIC the X-chromosomal ASE data was limited in case of multiple ASE sites to only one site per gene, by selecting the site with coverage >7 reads in the largest number of tissues, in order to have equal representation from each gene for downstream analyses.

### Assessing ASE across tissues

For GTEX-UPIC sample for which ASE data from up to 16 tissues per each ASE site was available, we applied the two-sided Hierarchical Grouped Tissue Model (GTM*) implemented in MAMBA 1.0.0^48,49^ to ASE data. The Gibbs sampler was run for 200 iterations with a burn-in of 50 iterations.

GTM* is a Bayesian hierarchical model that borrows information across tissues and across variants, and provides parameter estimates that are useful for interpreting global properties of variants. It classifies the sites into ASE states according to their tissue-wide ASE profiles and provides an estimate of the proportion of variants in each of the five different ASE states (strong ASE across all tissues (SNGASE), moderate ASE across all tissues (MODASE), no ASE across all tissues (NOASE), and heterogeneous ASE across tissues (HET1 and HET0)).

To summarize the GTM* output in the context of XCI, SNGASE was considered to reflect full XCI, MODASE and NOASE together to represent partial XCI with similar effects across tissues, and HET1 and HET0 to reflect partial yet heterogeneous patterns of XCI across tissues. In order to combine estimates from two ASE states, we summed the estimated proportions in each class, and subsequently calculated the 95% confidence intervals for each remaining ASE state using Jeffreys prior.

### Determining XCI status in GTEX-UPIC

In addition to the ASE states provided by the above MAMBA analysis, genic XCI status was assessed by comparing the allelic ratios at each X-chromosomal ASE site in each tissue individually. For each ASE site, the alleles were first mapped to Xa and Xi; the allele with lower combined relative expression across tissues was assumed the Xi allele. As an exception, at *XIST* the higher expressing allele was assumed the Xi allele. The significance of Xi expression at each ASE observation was tested using a one-sided binomial test, where the hypothesized probability of success was set at 0.025, i.e., the fraction of Xi expression from total expression was expected to be significantly greater than 0.025. To account for multiple testing, FDR correction was applied, using the qvalue R package, to the P-values from the binomial test for each of the 16 tissues separately. Observations with q-values < 0.01 were considered significant, i.e., indicative of incomplete XCI at the given ASE site and tissue.

### Biallelic expression in single cells

Biallelic expression in individual cells in the X chromosome was assessed only at ASE sites covered by the minimum of eight reads. A site was considered biallelically expressed when 1) allelic expression > 0.05, and 2) one-sided binomial test indicated allelic expression to be at least nominally significantly greater than 0.025. Only genes with at least two observations of biallelic expression across all cells within a sample were counted as biallelic.

### Phasing scRNA-seq data

We assigned each cell to either of two cell populations distinguished by the parental X-chromosome designated for inactivation utilizing genotype phasing. For the YRI samples, where parental genotype data was available, the assignment to the two parental cell populations was unambiguous for all cells where X-chromosomal sites outside PAR1 or frequently biallelic sites were expressed. For 24A no parental genotype data was available, and hence we utilized the correlation structure of the expressed X-chromosomal alleles across the 948 cells to infer the two parental haplotypes utilizing the fact that in individual cells the expressed alleles at the chrX sites subject to full inactivation (i.e. the majority chrX ASE sites), are from the X chromosome active in each cell (Supplementary Methods). In other words, while monoallelic expression in scRNA-seq in the autosomes is largely stochastic in origin, in the X chromosome the pattern of monoallelic expression is consistent across cells with the same parental X chromosome active^21^, unless a gene is expressed also from the inactive X. As such, for the phase inference calculations, we excluded all PAR1 sites and all additional sites that were frequently biallelic, to minimize the contribution of escape genes to the phase estimation. After assigning each informative cell to either of the parental cell populations, the reference and alternate allele reads for each ASE site were mapped to active and inactive allele reads within each sample using the actual or inferred parental haplotypes. The data was first combined per variant by taking the sum of active and inactive counts separately across cells, and further similarly combined per gene, if multiple SNPs per gene were available. For 24A the allele expressed at *XIST* was assumed the Xi allele, in line with the exclusive Xi expression in the Yoruba samples confirmed using the information on parental haplotypes.

### Determining XCI status from scRNA-seq ASE

Before calling XCI status using the Xa and Xi read counts from the phased data aggregated across cells, we excluded all sites without fewer than five cells contributing ASE data at each gene and also all sites with coverage lower than eight reads across cells within each sample. To determine whether the observed Xi expression is significantly different from zero, hence indicative of incomplete XCI at the site / gene, we required the Xi to total expression ratio to be significantly (q-value<0.01) greater than hypothesized upper bound for error, 0.025. This threshold was determined using the proportion of miscalled alleles at *XIST* ASE sites (by definition, *XIST* should express only alleles from the inactive chrX) in the two YRI samples, which presented with fully skewed XCI, i.e., the same active X chromosome across all assessed cells. Median proportion of miscalled *XIST* alleles was 0, yet one site in one of the samples showed up to 2.5% of other allele calls, and hence this was chosen as the error margin. FDR correction, conducted using the qvalue R package, was applied to each sample individually. Genes where at least one of the samples showed significant Xi expression were considered partially inactivated, while the remaining were classified as subject to full XCI. Allelic dropout, which is extensive in scRNA-seq^18,27^, can lead to biases in allelic ratios in individual cells, i.e., in our case resulting in false negatives where true escape genes are classified as inactivated, this approach utilized is based on using aggregate data across several cells and hence the XCI status estimates are robust to such errors.

### ChrX and chrY expression in PAR1

Using the parental origin of each allele reference and alternate allele read counts at PAR1 ASE sites were assigned to X and Y chromosomes (i.e. maternally and paternally inherited alleles, respectively). For each sample, the PAR1 ASE data was summarized by gene by taking the sum of X and Y chromosome reads across all informative ASE sites within each gene. Significance of deviation from equal expression was assessed using a two-sided binomial test.

### Manual curation of heterozygous variants from ASE analyses

Twenty-two heterozygous variants assessed in chrX ASE analysis were subjected to manual curation due to providing results in the XCI analysis that were in conflict with previous assignment of the underlying gene to be subject to full XCI. For each sample, BWA-aligned germline bams were viewed in IGV using either WGS or WES data. The presence of a number of characteristics called into question the confidence of the variant read alignments and thus the variant itself (Supplementary Methods). Allele balance that deviated significantly from 50:50 was considered suspect and often coincided with the existence of homology between the reference sequence in the region surrounding the variant and another area of the genome, as ascertained using the UCSC browser self-chain track and/or BLAT alignment of variant reads from within IGV. Other sequence-based annotations added to the VCF by HaplotypeCaller were also evaluated in the interests of examining other signatures of ambiguous mapping. The phasing of nearby variants was also considered. If phased variants occurred in the DNA sequencing data that were not assessed in the ASE analysis, those variants were considered suspect.

## Extended Data

**Extended Data Figure 1:**
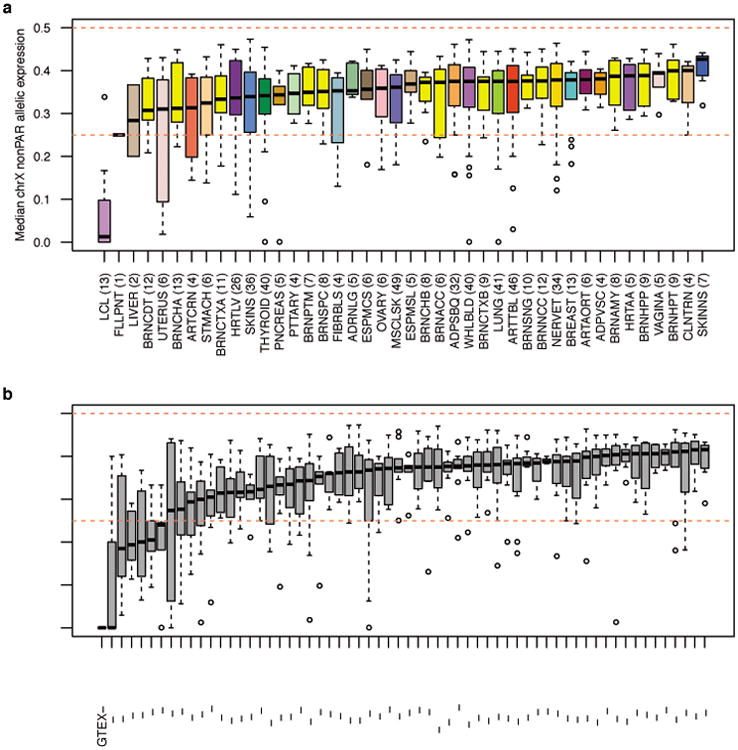
Assessment of skew in XCI in GTEx female samples (V3 analysis release) a) Shows the estimated skew in XCI by tissue across individuals and b) shows the skew in XCI by individual across tissue samples available. Number in brackets after tissue or sample name gives the number of individuals or tissues, respectively, contributing to each boxplot. Details of the analysis is given in the Supplementary Note.

**Extended Data Figure 2:**
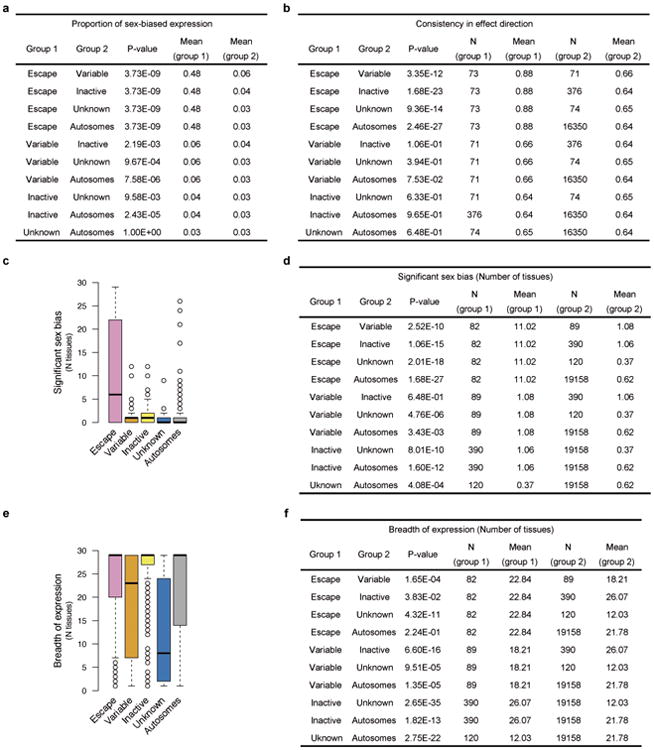
Comparison of expression characteristics between reported genic XCI categories in the GTEx data a) Table showing the statistics for the comparison of the proportion of significantly biased (FDR<1%) genes by reported XCI status. Distributions are illustrated in Fig. 2b. N = 29 for all comparisons. b) Table showing the statistics for the comparison of the consistency in effect sizes across tissues. Distributions are illustrated in Fig. 2c. Only genes expressed in at least five of the 29 tissues are included. c) Number of tissues showing significant sex bias (FDR<1%) per gene by reported XCI status. d) Statistics for the comparison illustrated in c). e) Number of tissues in which genes are expressed by reported XCI status. f) Statistics for the comparison illustrated in e). All P-values are from two-sided Wilcoxon tests, except for a) where paired, two-sided Wilcoxon test was applied. Only genes assessed for sex bias in at least one tissue are included unless otherwise stated.

**Extended Data Figure 3:**
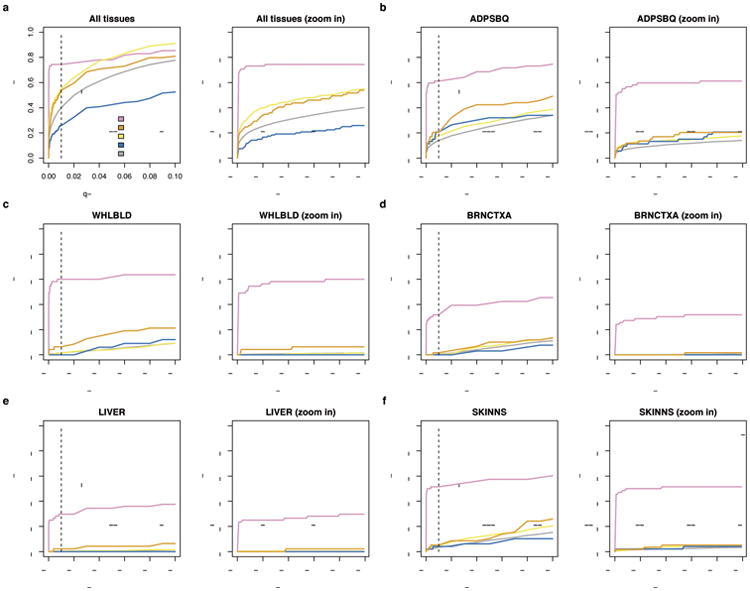
Change in the proportion of discovered sex-biased genes by XCI category with varying q-value cut-offs a) The proportion of sex-biased genes across tissues. Here a gene is classified as sex-biased if the q-value for association falls below the given threshold in at least one tissue. b-f) Examples of the change in the proportion of sex-biased expression in individual tissues. The dashed black line indicates the FDR<1% cut-off applied in the analyses to determine sex-biased expression.

**Extended Data Figure 4:**
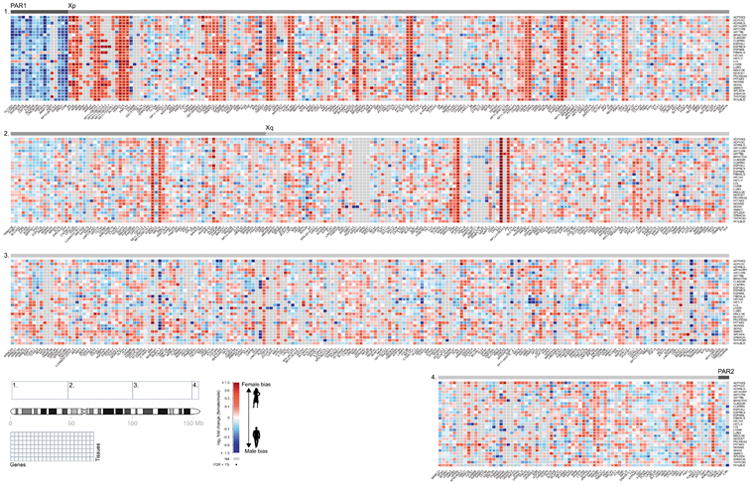
Heatmap representation of male-female expression differences in all assessed X-chromosomal genes (N=681) across 29 GTEx tissues The color scale displays the direction of sex bias with red color indicating higher female expression. Genes that were too weakly expressed in the given tissue type to be assessed in the sex bias analysis are colored grey. Dots mark the observations where sex bias was significant at FDR<1%

**Extended Data Figure 5:**
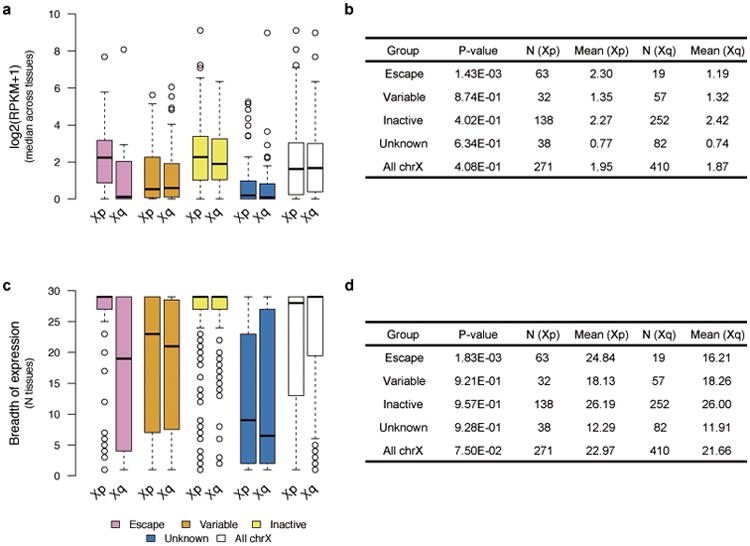
Comparison of expression characteristics between Xp and Xq, the evolutionary newer and older regions of chrX, respectively, by XCI status and for the whole chromosome a) and b) show level of median expression across GTEx tissues in log2 RPKM units, and c) and d) show the breadth of expression measured as the number of tissues (max = 29) in which genes are expressed (median expression across samples > 0.1 RPKM and expressed in more than 10 individuals at >1 counts per million). P-values are calculated using the Wilcoxon Rank Sum test. All genes expressed in at least one tissue are included in the comparisons.

**Extended Data Figure 6:**
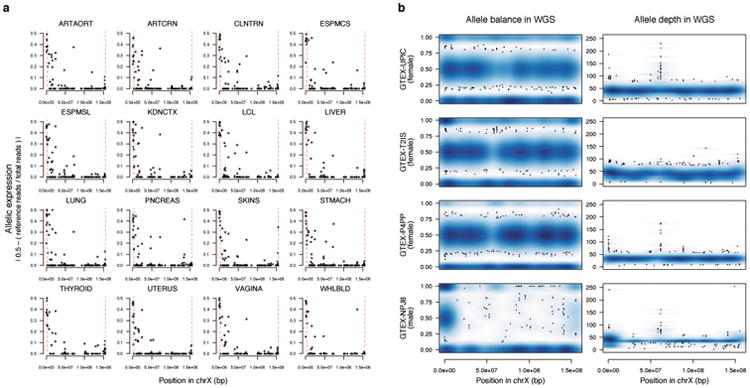
X-chromosomal RNA-seq and WGS data in the GTEx donor with fully skewed XCI (GTEX-UPIC) a) Allelic expression in chrX in 16 RNA-sequenced tissue samples available from the donor. Dashed red lines indicate PAR1 and PAR2 boundaries. b) Allele balance and allele depth across chrX in WGS for GTEX-UPIC and randomly chosen two female and one male GTEx WGS samples.

**Extended Data Figure 7:**
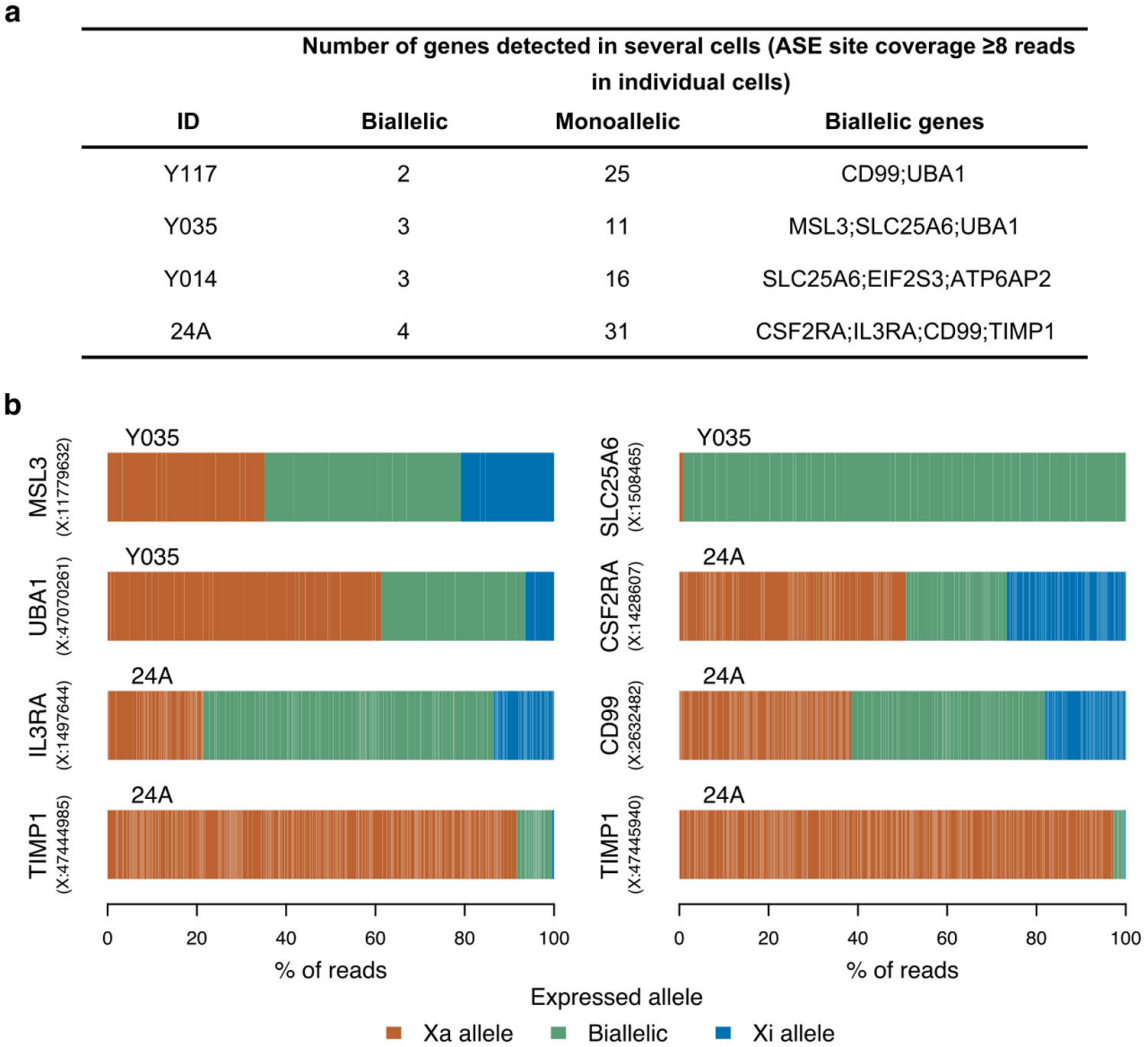
Expressed alleles at biallelically expressed ASE sites in scRNA-seq a) X-chromosomal genes repeatedly biallelic in scRNA-seq (see Methods for details). b) Illustration of the relative expression from the two alleles at all X-chromosomal ASE sites that were repeatedly biallelically expressed across cells in either of the two scRNA-seq samples that showed random XCI (Y035 and 24A). Narrow white lines separate observations from individual cells.

**Extended Data Figure 8:**
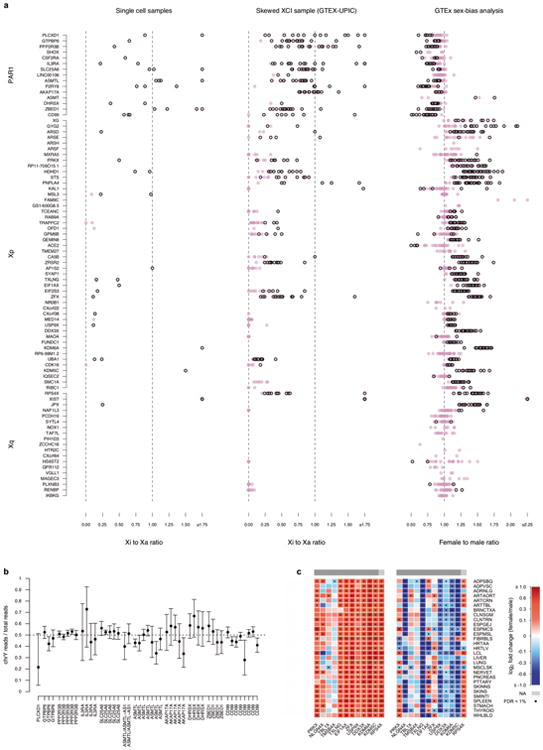
Assessment of the level of Xi expression at escape genes and in different regions of the X chromosome a) The ratio of Xi-to-Xa expression in the single cell samples (left panel; each circle represents a sample) and in the skewed XCI donor from GTEx (middle panel; each circle represents a tissue), and the female-to-male ratio in expression (right panel, each circle represents a tissue) at reported escape genes. Genes are ordered according to their location in the X chromosome with genes in the pseudoautosomal region residing in the top part of the figure. Dark border around a circle indicate there was significant evidence for Xi expression greater than the baseline in the given sample or tissue (left and middle panels) or significant sex-bias in the given tissue (right panel). Given some outliers, e.g. *XIST*, the Xi-to-Xa ratio is capped at 1.75 and female-to-male ratio at 2.25. b) The relative expression arising from the X and Y chromosome at PAR1 genes in skeletal muscle in eight males. The allelic expression at these genes was assigned to the two chromosomes utilizing parental genotypes available for these samples (see Methods for details). The dashed line at 0.5 indicates the point where expression from X and Y chromosomes is equal. The error bars give the 95% confidence intervals for the observed read ratio. c) Heatmap representation of the change in pattern of sex-bias at 13 X-Y homologous gene pairs (see Methods for details) in nonPAR from only including the X-chromosomal expression (heatmap on the left) to accounting for the Y-chromosomal expression (heatmap on the right). The color scale displays the direction of sex-bias with red color indicating higher female expression. Genes that were too lowly expressed in the given tissue type to be assessed in the sex-bias analysis are colored grey. Dots mark the observations where sex-bias was significant at FDR<1%. The grey bars on top of the heatmaps indicate the location of the gene in the X chromosome: dark grey indicating Xp and lighter grey Xq.

**Extended Data Table 1:** Tissues, individuals and genes in the GTEx sex-bias analysis

	Tissues	Individuals				Genes analyzed		
Abbreviation	Full name	All	Females	Males	Mean age	All	Autosomes	ChrX
ADPSBQ	Adipose - Subcutaneous	297	186	111	52.15	15,273	14,735	538
ADPVSC	Adipose - Visceral (Omentum)	184	117	67	51.97	15.301	14,765	536
ADRNLG	Adrenal Gland	126	70	56	50.51	14.956	14,435	521
ARTAORT	Artery - Aorta	197	126	71	51.11	14.675	14,137	538
ARTCRN	Artery - Coronary	118	70	48	51.7	14,881	14,350	531
ARTTBL	Artery - Tibial	284	183	101	50.26	14,501	13,981	520
BRNCTXA	Brain - Cortex	92	66	26	57.67	15,339	14,791	548
CLNSGM	Colon - Sigmoid	114	72	42	48.28	15,045	14,524	521
CLNTRN	Colon - Transverse	255	159	96	50.93	15,732	15,181	551
ESPGEJ	Esophagus - Gastroesophageal Junction	124	74	50	53.52	14,770	14,245	525
ESPMCS	Esophagus - Mucosa	169	97	72	48.89	15,137	14,617	520
ESPMSL	Esophagus - Muscularis	126	re	48	50.74	14,879	14,356	523
FIBRBLS	Cells - Transformed fibroblasts	240	150	90	50.2	13,635	13,158	477
HRTAA	Heart - Atrial Appendage	218	137	81	48.62	14,662	14,145	517
HRTLV	Heart - Left Ventricle	159	105	54	53.64	14,075	13,586	489
LCL	Cells - EBV-transformed lymphocytes	190	123	67	50.75	13,067	12,621	446
LIVER	Liver	96	63	33	53.52	14,031	13,556	475
LUNG	Lung	277	181	96	52.06	16,154	15,590	564
MSCLSK	Muscle - Skeletal	361	228	133	51.85	13,623	13,153	470
NERVET	Nerve - Tibial	256	163	93	51.65	15,563	15,020	543
PNCREAS	Pancreas	149	87	62	50.09	14,355	13,861	494
PTTARY	Pituitary	86	64	22	56.37	16,068	15,489	579
SKINNS	Skin - Not Sun Exposed (Suprapubic)	195	128	67	53.06	15,601	15,069	532
SKINS	Skin - Sun Exposed (Lower leg)	300	188	112	52.22	15,746	15,211	535
SMINTI	Small Intestine - Terminal Ileum	77	43	34	47.62	15,594	15,046	548
SPLEEN	Spleen	89	50	39	48.26	14,993	14,469	524
STMACH	Stomach	169	97	72	48.2	15,604	15,057	547
THYROID	Thyroid	278	179	99	52.14	15,974	15,417	557
WHLBLD	Whole Blood	338	213	125	51.64	13,187	12,751	436
Total		449	290	159	52.27	19,839	19,158	681

**Extended Data Table 2:** Single-cell RNA-seq samples

ID	24A	Y117	Y035	Y014
Ancestry	China, Asia	Yoruba / Nigeria, Africa	Yoruba / Nigeria, Africa	Yoruba / Nigeria, Africa
Design	Singleton	Trio	Trio	Trio
Genotype data	WES	WGS	WES	WES
Number of cells	742	96	48	48
Cell type	Dendritic cells	LCL	LCL	LCL
Sequenced read pairs (mean (range))	1,187,000 (335-7,403,000)	2,547,000 (38,190-5,126,000)	2,571,000 (46,940-5,038,000)	2,436,000 (69,130-5,457,000)
Aligned read pairs* (mean (range))	808,600 (197-5,727,000)	1,471,000 (14,910-3,309,000)	1,459,000 (16,400-2,893,000)	1,391,000 (14,920-3,067,000)
Alignment rate (mean (range))	0.667(0.271-0.799)	0.545(0.251-0.645)	0.551 (0.266-0.615)	0.526(0.175-0.606)
Skew in XCI (% maternal active : % paternal active)	54:46 (373 cells where one parental chromosome active, 315 cells where the other parental chromosome active, 54 cells uninformative for X- chromosomal phasing)	100:0 (90 cells where maternal X chromosome active, 6 cells uninformative for X- chromosomal phasing)	79:21 (37 cells where maternal X chromosome active, 8 cells where paternal X chromosome active, 2 cells uninformative for X- chromosomal phasing)	100:0 (43 cells where maternal X chromosome active, 2 cells uninformative for X- chromosomal phasing)
Notes	Due to the unavailability of parental genotype information, the parental origin of the inferred X- chromosomal haplotypes is unknown			

* uniquely aligned, properly paired, QC passed reads.

## Supplementary Material

reporting summary

supp_infoguide

supp_note

supp_table1

supp_table10

supp_table11

supp_table12

supp_table13

supp_table14

supp_table2

supp_table3

supp_table4

supp_table5

supp_table6

supp_table7

supp_table8

supp_table9

## Figures and Tables

**Figure 1:**
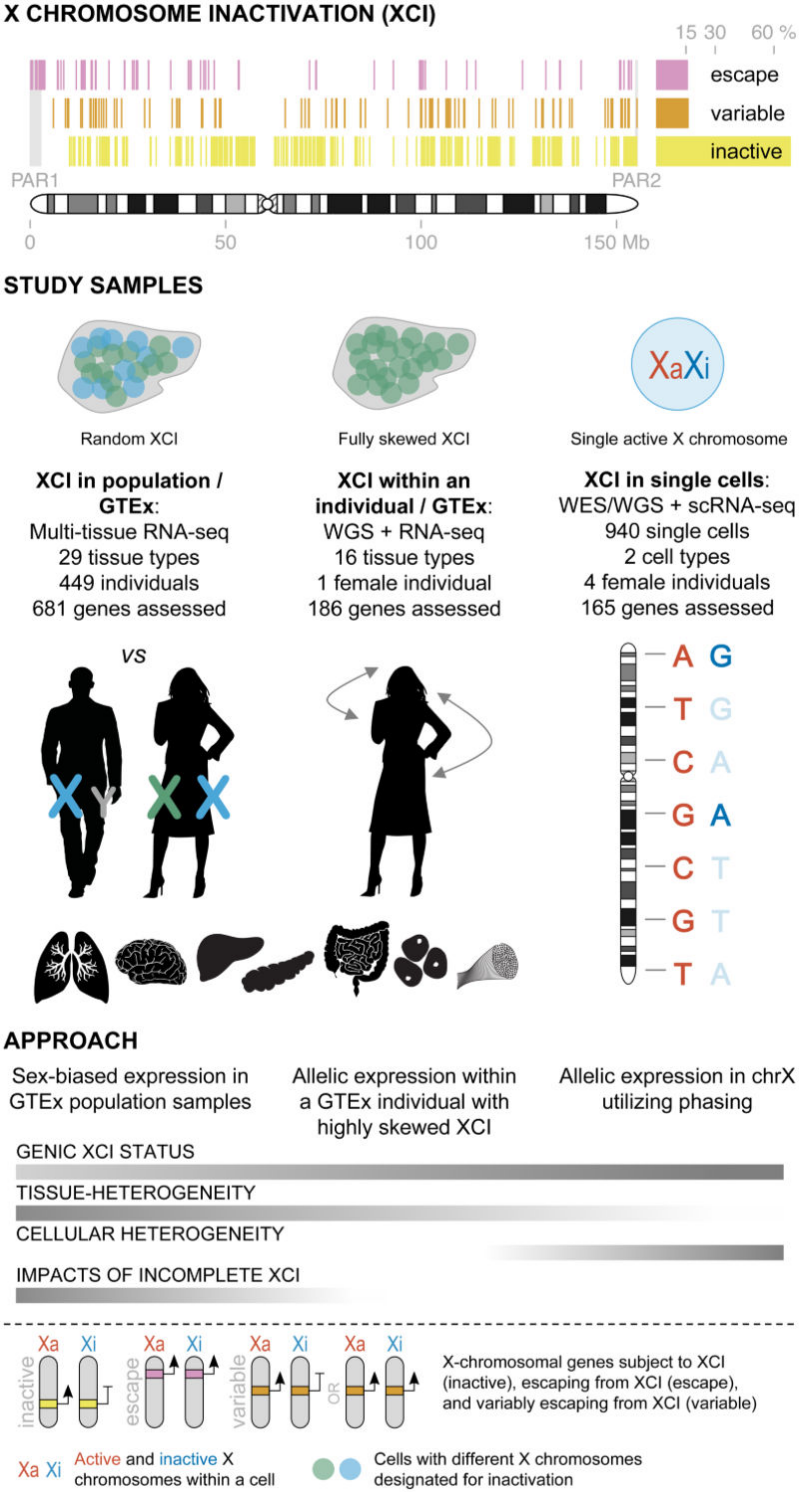
Schematic overview of the study. Previous expression-based surveys of XCI^1,2^ have established the incomplete and variable nature of XCI, but these studies have been limited in the tissue types and samples assessed. To investigate the landscape of XCI across human tissues, we combined three approaches: 1) sex biases in expression using population-level GTEx data across 29 tissue types, 2) allelic expression in 16 tissue samples from a female GTEx donor with fully skewed XCI, and 3) validation using single cell RNA-seq by combining allelic expression and genotype phasing. WGS, whole genome sequencing; WES, whole exome sequencing; scRNA-seq, single-cell RNA-seq.

**Figure 2:**
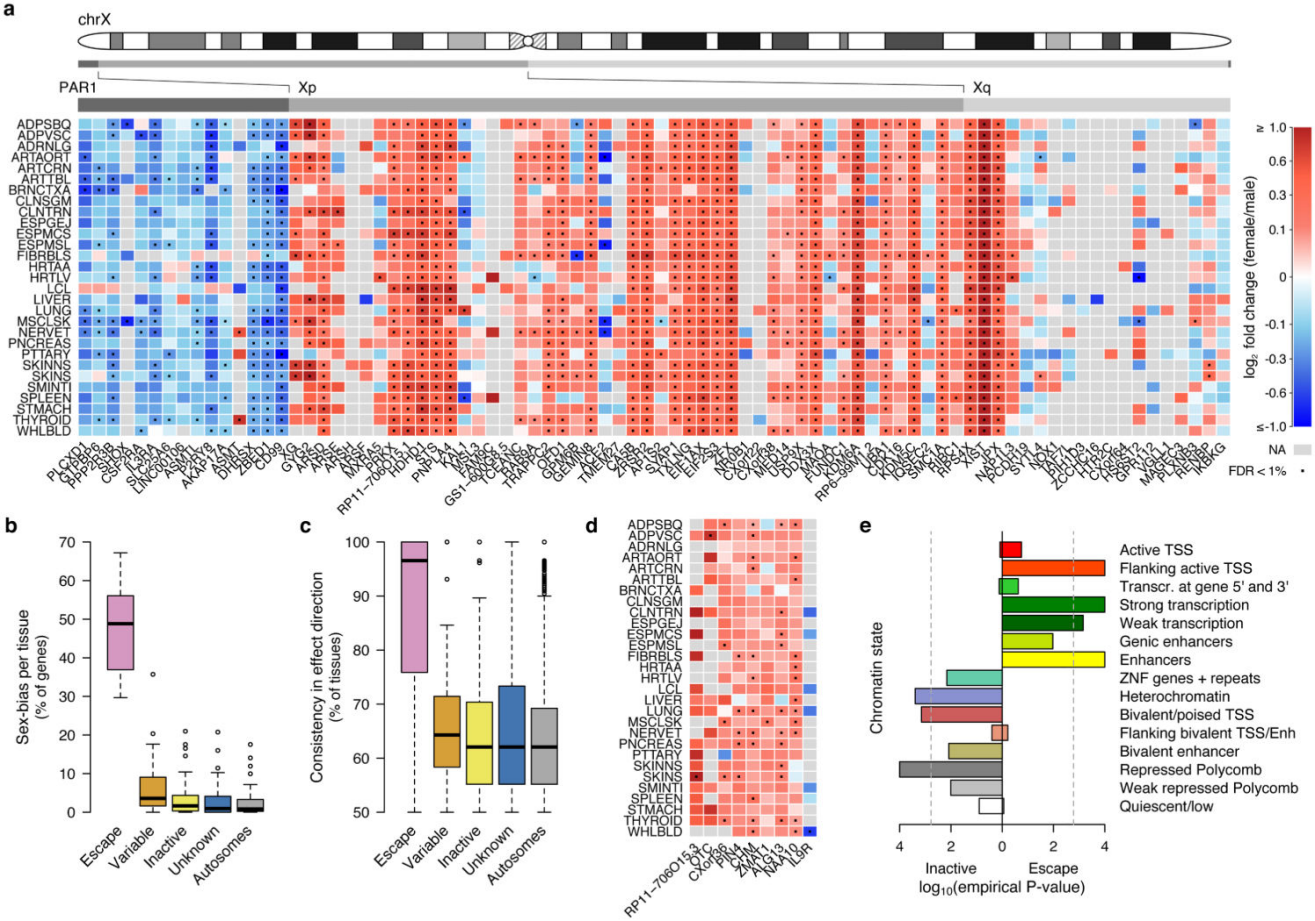
Assessment of tissue-sharing and population-level impacts of incomplete XCI in GTEx data. a) Male-female expression differences in reported XCI-escaping genes (N=82) across 29 GTEx tissues. b) Proportion of significantly biased (FDR<1%) genes in each tissue by reported XCI status. c) Proportion of tissues where the bias direction is shared by reported XCI status. Genes expressed in at least five tissues are included. d) Sex bias pattern of nine genes not classified as full escape genes that follow a similar profile to established escape genes. e) Chromatin state enrichment between escape and inactive genes in the Roadmap Epigenomics^46^ female samples.

**Figure 3:**
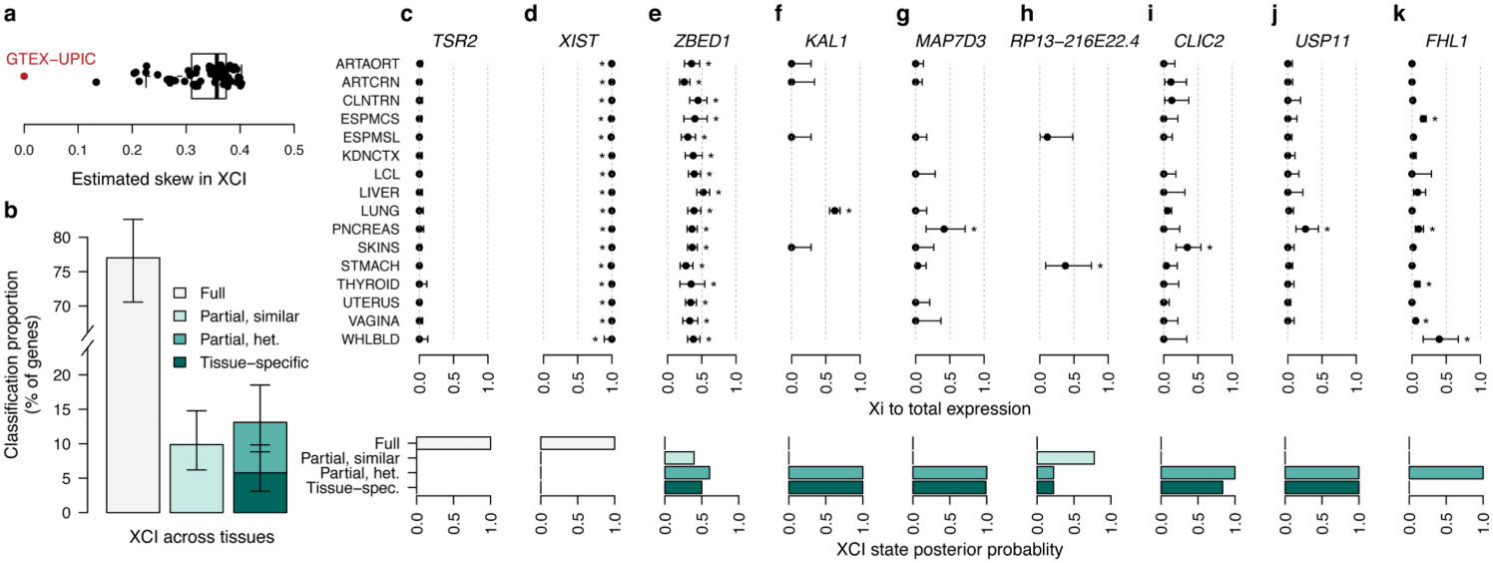
Assessment of tissue-sharing of XCI in a GTEx donor with highly skewed XCI. a) Distribution of the skewness of XCI in GTEx female samples (N=62, V3 release). Each data point shows the mean skew in XCI across tissue samples per individual. b) Classification of X-chromosomal genes (N=186) into full or incomplete and tissue-shared or heterogeneous XCI based on the analysis of ASE patterns across tissues. Error bars show the 95% credible interval. c-e) Examples of genes where the ASE-based assessment of XCI status match previously reported assignments (*TSR2*, inactive; *XIST*, escape; *ZBED1*, escape). Note that *XIST* is, unusually for an escape gene, expressed monoallelically, only from Xi. f) *KAL1* shows strong evidence for tissue-specific escape. g-k) Genes without previous or conclusive evidence for escape from XCI but classified as incompletely inactivated in this sample. In c-k asterisks indicate that the Xi expression in the given tissue was significant at FDR < 1% (one-sided binomial test) and errors bars show the 95% confidence interval.

**Figure 4:**
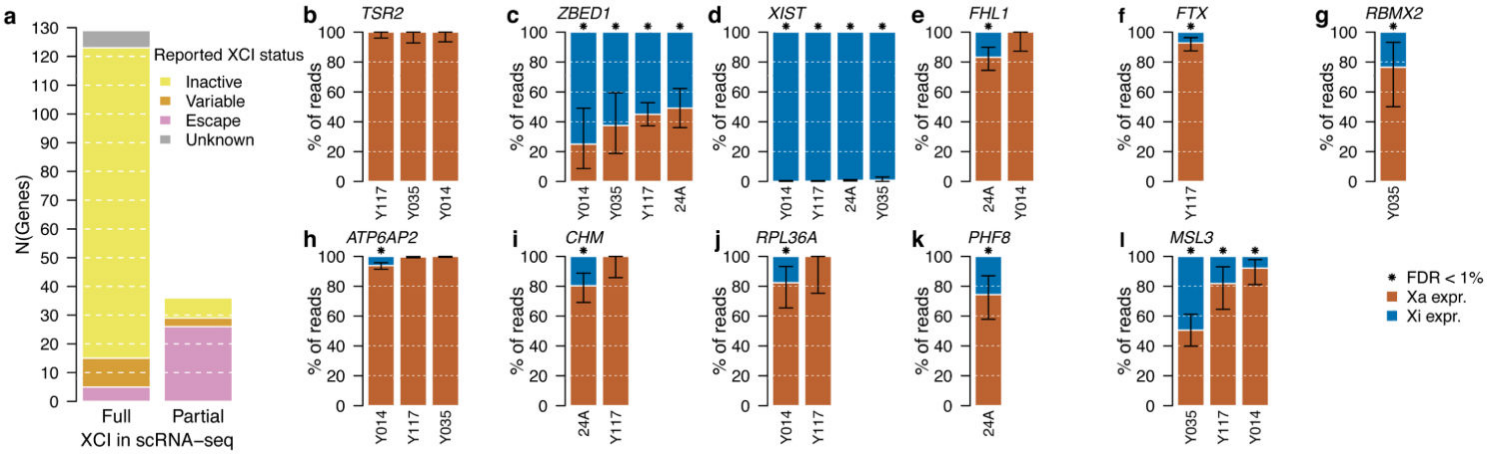
Analysis of XCI using scRNA-seq. a) Proportion of genes demonstrating full and partial XCI in the ASE analysis in single cell RNA-seq data, and the concordance with previously reported XCI status. b-l) Examples of genes with different XCI patterns in scRNA-seq: previously reported inactive gene (b), known escape gene in PAR1 (c), escape gene with known exclusive expression from Xi (d) new candidates for escape genes that demonstrate incomplete XCI in only a subset of samples (e-k), and a known escape gene that shows escape of varying degrees in the three samples (Pearson's Chi-squared test for equal proportions, P=3.80×10^-7^) (l). Asterisk above a bar indicates that the proportion of Xi expression, i.e. blue bar, in a given sample is significantly greater than the expected baseline (FDR < 1%, one-sided binomial test). Error bars show the 95% confidence interval.
