# Supplementary material for: Landscape of X chromosome inactivation across human tissues
Source: Nature. Author manuscript; Available in PMC 2018 Apr 11. (PMC5685192; doi:10.1038/nature24265)
Supplement: reporting summary [file NIHMS905235-supplement-reporting_summary.pdf]

nature research

Corresponding author(s): Tukiainen / MacArthur

☐ Initial submission ☐ Revised version ☒ Final submission

Life Sciences Reporting Summary

Nature Research wishes to improve the reproducibility of the work that we publish. This form is intended for publication with all accepted life science papers and provides structure for consistency and transparency in reporting. Every life science submission will use this form; some list items might not apply to an individual manuscript, but all fields must be completed for clarity.

For further information on the points included in this form, see Reporting Life Sciences Research. For further information on Nature Research policies, including our data availability policy, see Authors & Referees and the Editorial Policy Checklist.

▸ Experimental design

1. Sample size

Describe how sample size was determined.

No statistical methods were used to predetermine sample size.

2. Data exclusions

Describe any data exclusions.

The GTEx samples were curated according to pre-established QC criteria as detailed in the accompanying manuscript by Aguet et al. ScRNA-seq data was limited to those cells that were informative for chromosome X allelic expression.

3. Replication

Describe whether the experimental findings were reliably reproduced.

The analyses conducted were exploratory and the results were not replicated in independent data sets. However each analysis included multiple data points (individuals and/or tissues) thus providing further support for the conclusions drawn.

4. Randomization

Describe how samples/organisms/participants were allocated into experimental groups.

The experiments were not randomized. The study included no allocation into experimental groups.

5. Blinding

Describe whether the investigators were blinded to group allocation during data collection and/or analysis.

The investigators were not blinded to allocation during experiments and outcome assessment. The study included no allocation into experimental groups.

Note: all studies involving animals and/or human research participants must disclose whether blinding and randomization were used.

6. Statistical parameters

For all figures and tables that use statistical methods, confirm that the following items are present in relevant figure legends (or in the Methods section if additional space is needed).

n/a	Confirmed	
☐	☒	The exact sample size (*n*) for each experimental group/condition, given as a discrete number and unit of measurement (animals, litters, cultures, etc.)
☐	☒	A description of how samples were collected, noting whether measurements were taken from distinct samples or whether the same sample was measured repeatedly
☒	☐	A statement indicating how many times each experiment was replicated
☐	☒	The statistical test(s) used and whether they are one- or two-sided (note: only common tests should be described solely by name; more complex techniques should be described in the Methods section)
☐	☒	A description of any assumptions or corrections, such as an adjustment for multiple comparisons
☐	☒	The test results (e.g. *P* values) given as exact values whenever possible and with confidence intervals noted
☐	☒	A clear description of statistics including central tendency (e.g. median, mean) and variation (e.g. standard deviation, interquartile range)
☐	☒	Clearly defined error bars

See the web collection on statistics for biologists for further resources and guidance.

Software

Policy information about availability of computer code

7. Software

Describe the software used to analyze the data in this study.	RNA-seq alignment: Tophat version v1.4.1, STAR versions 2.4.2a, 2.4.1a or 2.3.0e; RNA-seq QC and quantification: RNA-SeQC; Allelic expression and variant calling: GATK version 3.1 or 3.4. Data processing: R version 3.4.0.

For manuscripts utilizing custom algorithms or software that are central to the paper but not yet described in the published literature, software must be made available to editors and reviewers upon request. We strongly encourage code deposition in a community repository (e.g. GitHub). *Nature Methods* guidance for providing algorithms and software for publication provides further information on this topic.

Materials and reagents

Policy information about availability of materials

8. Materials availability

Indicate whether there are restrictions on availability of unique materials or if these materials are only available for distribution by a for-profit company.	All unique materials are readily available from the authors or from commercial sources as described in the Online Methods.

9. Antibodies

Describe the antibodies used and how they were validated for use in the system under study (i.e. assay and species).	The antibody panels used to enrich for all known blood DC population for single cell sorting and single cell RNA-sequencing (scRNA-seq) are described in Villani et al (Science 2017). All antibodies are commercially available as described in Supplementary Table 14.

10. Eukaryotic cell lines

a. State the source of each eukaryotic cell line used.	YRI LCLs were obtained from NHGRI Sample Repository for Human Genetic Research (Coriell Institute for Medical Research).
b. Describe the method of cell line authentication used.	None of the cell lines used were authenticated.
c. Report whether the cell lines were tested for mycoplasma contamination.	Coriell Biorepositories declares that their lymphoblastoid cell lines are free of bacterial, fungal or mycoplasma contamination. No other tests were run to test for mycoplasma contamination.
d. If any of the cell lines used are listed in the database of commonly misidentified cell lines maintained by ICLAC, provide a scientific rationale for their use.	No commonly misidentified cell lines were used.

Animals and human research participants

Policy information about studies involving animals; when reporting animal research, follow the ARRIVE guidelines

11. Description of research animals

Provide details on animals and/or animal-derived materials used in the study.	No animals were used

Policy information about studies involving human research participants

12. Description of human research participants

Describe the covariate-relevant population characteristics of the human research participants.	24A: Female, Asian ancestry, 25 yo, healthy<br>Y117, Y035 and Y014: Female, African ancestry, age and health status unknown<br>GTEx-UPIC: Female, European ancestry, 21 yo, cause of death asphyxiation<br>See Extended Data Table for information on other GTEx donors
