# Supplementary material for: Landscape of X chromosome inactivation across human tissues
Source: Nature. Author manuscript; Available in PMC 2018 Apr 11. (PMC5685192; doi:10.1038/nature24265)
Supplement: supp_infoguide [file NIHMS905235-supplement-supp_infoguide.doc]

**SI Guide**

1. File titles

Merged pdf:

Supplementary Methods; Supplementary Table legends and Supplementary Table 14; Supplementary Discussion; Supplementary Note

Separate files:

Supplementary Table 1

Supplementary Table 2

Supplementary Table 3

Supplementary Table 4

Supplementary Table 5

Supplementary Table 6

Supplementary Table 7

Supplementary Table 8

Supplementary Table 9

Supplementary Table 10

Supplementary Table 11

Supplementary Table 12

Supplementary Table 13

2. File summaries

Merged pdf: This file contains more detailed description of methods, supplementary discussion, and a supplementary note describing the analysis of skew in XCI in GTEx female samples.

Supplementary Table 1. XCI status list compiled from previous studies.

Supplementary Table 2. Sex bias results for chrX from the GTEx analysis.

Supplementary Table 3. Nine genes that have not conclusively been described as escape genes in previous studies but follow a similar expression profile to escape genes in the GTEx sex bias analysis.

Supplementary Table 4: Variant QC for ASE.

Supplementary Table 5. X-chromosomal ASE results across 16 tissues in GTEX-UPIC.

Supplementary Table 6. Posterior probabilities for different ASE states for each X-chromosomal ASE site expressed in at least two tissues.

Supplementary Table 7. Association between posterior probabilities for different ASE states and XCI categories.

Supplementary Table 8. All scRNA-seq results for chrX.

Supplementary Table 9. Summary of observed XCI status in genes assessed in scRNA-seq samples.

Supplementary Table 10. Concordance of XCI status assignments from scRNA-seq with previous assignments.

Supplementary Table 11. New escape genes from scRNA-seq.

Supplementary Table 12. Xa and Xi expression between two X-chromosomal haplotypes in single cells. Supplementary Table 13. A summary of XCI analyses across the three data sets.
