# Supplementary material for: Landscape of X chromosome inactivation across human tissues
Source: Nature. Author manuscript; Available in PMC 2018 Apr 11. (PMC5685192; doi:10.1038/nature24265)
Supplement: supp_note [file NIHMS905235-supplement-supp_note.pdf]

Supplementary Information

Supplementary Methods

Single cell data preparation and sequencing

Flow cytometry staining and single cell sorting

For profiling of healthy DCs, peripheral blood mononuclear cells (PBMCs) were first isolated from fresh blood within 2hrs of collection, using Ficoll-Paque density gradient centrifugation as previously described[1]. Single-cell suspensions were stained per manufacturer recommendations with an antibody panel (Supplementary Table 14) designed to enrich for all known blood DC population for single cell sorting and single cell RNA-sequencing (scRNA-seq) profiling. PBMCs cell suspension was first immunostained with an antibody cocktail (CD3, CD19, CD56, CD14) to exclude other blood lineages (LIN), and with antibodies for known DC markers (HLA-DR, CD11C, CD1C, CD141, CD123; Supplementary Table 14). Since CD14–CD16+ cells within human LIN–HLA-DR+ fraction has been classified as both monocytes and DCs, we only excluded CD14+ monocytes using a stringent gate. Briefly, DCs were defined as live, lineage (LIN: CD3,CD19,CD56)–CD14–HLA-DR+ cells. Conventional DCs (cDCs) were further defined as CD11C+ and 3 loose overlapping gates were drawn as an enrichment strategy to ensure a comprehensive and even sampling of both rare and common DC populations: CD11C+CD141+ (CD141; turquoise), CD11C+CD1C+ (CD1C; orange), CD11C+CD141-CD1C- ('Double Negative'; blue). Plasmacytoid DCs (pDCs) were defined as CD11C-CD123+ (pDC; purple). 24 single cells from four loosely gated populations (i.e. LIN–CD14–HLA-DR+CD11C–CD123+, LIN-CD14–HLA-DR+CD11C+CD141+, LIN–CD14–HLA-DR+CD11C+CD1C+, LIN–CD14–HLA-DR+CD11C+CD141–CD1C–) were sorted per 96-well plate, with each well containing 10ul of lysis buffer. A total of eight plates were analysed by single-cell RNA-sequencing.

All LCL cell lines were cultured according to Coriell's recommendation (medium: RPMI 1640, 2mM L-glutamine, 15% fetal bovine serum (all three from ThermoFisher Scientific)) in T25 tissue culture flask with 10-20 ml medium at 37°C under 5% carbon dioxide. Cells were split upon reaching cell density of approximately 300,000-400,000 viable cells/ml. All three lymphoblast cultures were split once prior to proceeding with single cell sorting. Cells were washed with 1X PBS, pellet resuspended and stained with DAPI (Biolegend) for viability according to manufacturer's recommendation.

All single live cells (for both DCs and LCL cell lines) were sorted in 96-well full-skirted eppendorf plate chilled to 4°C, pre-prepared with 10µl TCL buffer (Qiagen) supplemented with 1% beta-mercaptoethanol (lysis buffer) using BD FACS Fusion instrument. Single-cell lysates were sealed, vortexed, spun down at 300g at 4°C for 1 minute, immediately placed on dry ice and transferred for storage at -80°C.

Single-cell RNA-seq: Reverse transcription

Smart-Seq2 protocol was performed on single sorted cells as described[2,3], with some modifications. 748 single DCs isolated from healthy Asian female individual, along with 96 single cells from GM19240, 48 single cells from GM19199, and 48 single cells from GM18518 were profiled as described below. Single-cells lysates were thawed on ice for 2 minutes, then

centrifuged at 2,500rpm at 4°C for 1 minute. Lysates were mixed with 22μL (2.2X) of Agencourt RNAClean XP SPRI beads (Beckman-Coulter) and incubated at room temperature for 10 min. The lysate plate was transferred to a magnet (DynaMag-96 Side Skirted Magnet, Life Technologies), the supernatant was removed, and the beads were washed three times in 100μL of 80% ethanol, with care being taken to avoid loss of beads during the washes. Ethanol was removed, and the beads were left to dry at room temperature for 10 min. Beads were resuspended in 4μL of Elution Mix (1μL 10μM RT primer [5'AGACGTGTGCTCTTCCGATCT(T)30VN-3', IDT], 1μL 10 mM dNTP [Agilent], 0.1μL SUPERase•In RNase-Inhibitor [20 U/μL, Life Technologies], and 1.9μL nuclease-free water). The samples were denatured at 72° C for 3 min and placed immediately on ice afterwards. 7μL of the Reverse Transcription Mix was subsequently added (2μL 5x RT buffer [Thermo Scientific], 2μL 5 M Betaine [Sigma-Aldrich], 0.9μL 100mM MgCl2 [Sigma-Aldrich], 1μL 10μM TSO [5'- AGACGTGTGCTCTTCCGATCTNNNNNrGrGrG-3', IDT], 0.25 μL SUPERase•In RNase-Inhibitor [20U/μL, Life Technologies], 0.1μL Maxima H Minus Reverse Transcriptase [200U/μL, Thermo Scientific], and 0.75μL nuclease-free water). Every well was mixed with the resuspended beads. Reverse transcription was carried out by incubating the plate at 50°C for 90 min, followed by heat inactivation at 70°C for 10 min.

Single-cell RNA-seq: PCR pre-amplification

14μL of PCR Mix was added for a final PCR reaction volume of 25μL (0.5μL 10μM PCR primer [5'AGACGTGTGCTCTTCCGATCT-3', IDT], 12.5μL 2x KAPA HiFi HotStart ReadyMix [KAPA Biosystems], 1μL nuclease-free water). The reaction was carried out with an initial incubation at 98°C for 3 min, followed by 22 cycles at (98°C for 15 sec, 67°C for 20 sec, and 72°C for 6 min) and a final extension at 72°C for 5 min. PCR products were purified by mixing with 20μL (0.8X) Agencourt AMPureXP SPRI beads (Beckman-Coulter), followed by incubation for 6 minutes at room temperature. The plate was placed on a magnet for 6 minutes, the supernatant was removed, and the beads were washed twice with 100μL of 70% ethanol, with care being taken to avoid loss of beads during the washes. Ethanol was removed, and the beads were left to dry at room temperature for 10 min. The beads were resuspended in 20μL TE buffer (Teknova). The plate was placed on the magnet and supernatant containing the amplified cDNA was transferred to a new 96-well PCR plate. The cDNA SPRI clean-up was repeated a second time to remove all residual primer dimers following the same approach. The concentration of amplified cDNA was measured on the Synergy H1 Hybrid Microplate Reader (BioTek) using High-Sensitivity Qubit reagent (Life Technologies), and the size distribution of select wells was checked on a High-Sensitivity Bioanalyzer Chip (Agilent). Expected quantification was around 0.5-2 ng/μL with size distribution sharply peaking around 2kb.

Single-cell RNA-seq: Library preparation

Library preparation was carried out using the Nextera XT DNA Sample Kit (Illumina) with custom indexing adapters, allowing up to 384 libraries to be simultaneously generated in a 384-well PCR plate (note that DCs were processed in 384-well plate while LCL were processed in 96-well plate format). For each library, the amplified cDNA was normalized to 0.15-0.20ng/μL. The tagmentation reaction consisted of 0.625μL of cDNA mixed with 1.25μL Tagment DNA Buffer and 0.625μL Tagment DNA enzyme mix. The 2.5μL reaction was incubated at 55°C for 10 min and placed immediately on ice afterwards. The reaction was quenched with 0.625μL Neutralize Tagment Buffer and incubated at room temperature for 10 min. The libraries were amplified by

adding 1.875 µL Nextera PCR Master Mix, 0.625µL of 10µM i5 adapter (5'-AATGATACGGCGACCACCGAGATCTACAC[i5]TCGTCGGCAGCGTC-3', IDT, where [i5] signifies the 8 bp i5 barcode sequence (see below for sequences), and 0.625µL of 10µM i7 adapter (5'CAAGCAGAAGACGGCATACGAGAT[i7]GTGACTGGAGTTCAGACGTGTGCTCTTCCGATCTGGG-3', IDT, where [i7] signifies the reverse-complement of the 8 bp i7 barcode sequence (see below for sequences). The PCR was carried out at an initial incubation at 72°C for 3 min, 95°C for 30 sec, followed by 12 cycles of (95°C for 10 sec, 55°C for 30 sec, 72°C for 1 min), and a final extension at 72°C for 5 min. Following PCR amplification, 2.5µL of each library were pooled in a 2.0 mL microcentrifuge tube. The pool was mixed with 216µL (0.9X for 2.5ul of 96 cells pooled together (for LCL); 0.9X for 2.5ul of 384 cells pooled together (for DCs)) Agencourt AMPureXP SPRI beads (Beckman-Coulter) and incubated at room temperature for 5 min. The pool was placed on a magnet (DynaMag-2, Life Technologies) and incubated for 5 min. The supernatant was removed and the beads were washed twice in 1mL of 70% ethanol. The ethanol was removed and the beads left to dry at room temperature for 10 min. The beads were resuspended in 50µL of nuclease-free water. The tube was returned to the magnet, and the supernatant was transferred to a new 1.5mL microcentrifuge tube. The SPRI clean-up of the library was repeated a second time to remove all residual primer dimers. The concentration of the pooled libraries was measured using the High-Sensitivity DNA Qubit (Life Technologies), and the size distribution measured on a High-Sensitivity Bioanalyzer Chip (Agilent). Expected concentration of the pooled libraries was 10-30 ng/µL with size distribution of 300-700bp. For the DCs, we created pools of 384 cells, while 96 LCL samples were pooled at the time. We sequenced one library pool per lane as paired-end 25 base reads on a HiSeq2500 (Illumina).

Barcodes used for 96-well plate comprising GM19240:
i5 barcodes: AGGATCTA, AGGTTATC, ATTCCTCT, CAACTCTC, CATGCTTA, CCAACATT, CTAACTCG, CTACCAGG, CTGCGGAT, GGTCCAGA, GTCTGATG, TCTGGCGA
i7 barcodes: AGAACATT, AGTGTCTT, ATCCGACA, CAAGGCGA, GAATTGCT, GACCGAGA, GTCAAGTT, GTCTTAGT
Barcodes used for 96-well plate comprising GM19199 and GM18518:
i5 barcodes: AGGATCTA, AGGTTATC, ATTCCTCT, CAACTCTC, CATGCTTA, CCAACATT, CTAACTCG, CTACCAGG, CTGCGGAT, GGTCCAGA, GTCTGATG, TCTGGCGA
i7 barcodes: AACATAAT, AAGCAACT, AGGATGTG, GACGCTAT, TCAACTGT, TCCATGCT, TCGCACCT, TTGATAAT
Barcodes used for 384-well plate comprising single DCs:
i5 barcodes: AAGTAGAG, ACACGATC, TGTTCCGA, CATGATCG, CGTTACCA, TCCTTGGT, AACGCATT, ACAGGTAT, AGGTAAGG, AACAATGG, ACTGTATC, AGGTCGCA, GGTCCAGA, CATGCTTA, AGGATCTA, TCTGGCGA, AGGTTATC, GTCTGATG, CCAACATT, CAACTCTC, ATTCCTCT, CTAACTCG, CTGCGGAT, CTACCAGG
i7 barcodes: CTACCAGG, CATGCTTA, GCACATCT, TGCTCGAC, AGCAATTC, AGTTGCTT, CCAGTTAG, TTGAGCCT, ACCAACTG, GGTCCAGA, GTATAACA, TTCGCTGA, AACTTGAC, CACATCCT, TCGGAATG, AAGGATGT

Catalogue of X-inactivation status

In order to compare results from the ASE and GTEx analyses with previous observations on genic XCI status we collated findings from two earlier studies[4,5] that represent systematic expression-based surveys into XCI each study cataloguing hundreds of X-linked genes and together the data spanning two tissue types.

Data from Carrel&Willard

Carrel and Willard[4] presented the first comprehensive profile of XCI statuses for X-linked genes by comparing X-linked gene expression between human/rodent somatic cell hybrids containing either human Xa or Xi. In total they surveyed in total 624 X-chromosomal transcripts expressed in primary fibroblasts, including 471 transcripts annotated in NCBI build 34.3 and an additional 153 transcripts (including full-length mRNAs and ESTs) not associated with annotated genes, in nine cell hybrids each containing a different human Xi. Given the old genome build utilized and the large number of unannotated transcripts surveyed in the Carrel and Willard study, the reported gene names or chromosomal positions were not utilized in mapping the XCI status data the to current reference, but the primer pair sequences designed to test the expression of the transcripts (primers given in Table S9 in Carrel and Willard) were used to find the genomic location and gene corresponding to each transcript.

To perform in silico PCR against a comprehensive set of possible templates, we created three reference databases against which to align primers: 1) fully spliced transcripts, 2) unspliced transcripts, and 3) full genome. All were based on the hg19 genome and the Gencode v19 "Basic Set". Primer sequences were aligned using in-house software (unpublished), which retained any ungapped alignment with two or fewer mismatches, with at least one segment of five consecutive perfect matches, and with no mismatches in the five 3'-most primer bases. Results were screened for each primer pair, reporting any pairs of alignments (regardless of participating primers) consistent with the generation of an amplicon <=10kb in size.

In order to find the gene from Gencode v19 annotations corresponding each primer pair, the alignments to the three possible templates were dissected by first prioritizing alignments to fully spliced transcripts, then alignments to the unspliced transcripts and finally including alignments to hg19 search and manually curating the results in case of multiple reported and/or conflicting alignments.

Carrel and Willard also provided XCI statuses for 10 genes not surveyed in their study but reported in earlier studies. In the lack of primer sequence information, the transcripts were matched to Gencode v19 using reported transcript names. For 8 of these 10 genes the transcript name matched unambiguously gene name in the Gencode reference or a known alias, the two other transcripts (FLJ41633 and Hs.522028) were excluded.

In total 553 transcripts primer pairs (87% of the original 634 transcripts, which includes the ten previously surveyed genes) were successfully matched to X-chromosomal Gencode v19 reference mapping together to 470 unique chrX genes. 403 genes were represented by one primer sequence only and the corresponding Xi data was included as such, but for the 67 genes with alignments from multiple primers (53 genes with two aligned primers, 12 genes with three aligned primers, 2 genes (*USP9X* and *JPX*) with four aligned primers) data for Xi expression was averaged over the primers aligning to each gene. The level of Xi expression for individual primers within a gene were generally in good agreement (e.g. for *APS1S2* all three transcripts AP1S2,

Hs.121592, Hs.431654 showed Xi expression in all nine cell lines) with the exception of *SRPK3* for which the two aligned transcripts (PLXNB3 and STK23) gave completely discordant results. The 470 X-chromosomal genes in the final list were split into three XCI status categories based on the level of Xi expression (i.e. here the number of cell lines expressing the gene from Xi)

1. Gene was considered inactive if fewer than 25% of cell lines had detectable Xi expression: 344 genes (73%)
2. Gene was considered variable escape if 25-75% of cell lines had detectable Xi expression: 51 genes (11%)
3. Gene was considered escape more than 75% of cell lines had detectable Xi expression: 75 genes (16%)

<u>Data from Cotton et al</u>

Cotton et al.[5] surveyed XCI using allelic imbalance in clonal or near-clonal female LCL and fibroblast cell lines and provided XCI statuses for 508 genes (Additional file 7 / Table S5 in Cotton et al.) which were classified into three XCI status categories based on the level of expression from the suspected inactive allele (i.e. the allele with lower expression): escape (N=68), variable escape (N=146), and subject (N=294). To match this data with Gencode v19 annotations the reported gene name was compared with the Gencode “gene_name”, which yielded a unique match for 473 genes. For the remaining 35 genes for which the reported gene name did not match any of the gene names in the Gencode annotation file, the gene names were manually curated by searching for matching names from previous HGNC symbols or known aliases, and subsequently mapped to Gencode annotations using the updated gene names. After excluding *CXorf59*, which after updating the gene names matched to *CXorf22* already present in the data (both genes classified as “escape”), and *TMSL3* (classified as “escape” in Cotton et al), which similarly after updating the gene names matched to *TMSB4XP8* in chromosome 22, XCI statuses were available for 506 X-chromosomal genes.

<u>Combining lists</u>

To create a joint set of XCI statuses the two XCI gene lists were compared using the three categories (escape, variable escape and inactive (i.e. subject)). 345 genes were available in both lists, and of these XCI statuses agreed between the studies for 224 genes (23 escape, 13 variable escape and 188 inactive genes). For the remaining 121 genes for which the XCI statuses failed to align the following rules were applied to determine the XCI status in the combined list: 1) A gene was considered “escape” if it was called escape in one study and variable in the other (N=26), 2) “variable escape” if classified as escape and inactive (N=19), and 3) “inactive” if classified as inactive in one study and variable escape in the other (N=76). For the 287 genes unique to either of the study (125 genes from Carrel and Willard, 161 genes from Cotton et al), the XCI status given in the original study was adopted. The final combined list of XCI statuses thus consisted of 631 X-chromosomal genes including 99 escape (16%), 101 variable escape (16%) and 431 inactive (68%) genes.

Analysis of sex-biased expression

Differential expression analyses were conducted to identify genes that are expressed at significantly different levels between male and female samples using the GTEx V6 tissues with

RNA-seq and genotype data available from more than 70 individuals after excluding samples flagged in QC by the GTEx Laboratory, Data Analysis, and Coordinating Center. Given the strong correlation of expression profiles in the different brain subregions[6], only one brain region (BRNCTXA, Brain - Cortex) was included. Additionally, sex-specific tissues were excluded. Previous analyses point to breast tissue being an extreme outlier in terms of sex-biased expression[6], likely due to differences in tissue composition between the sexes. As such excess sex bias can hamper the detection of the sex biases in transcription related to XCI, breast tissue was also excluded from the XCI analyses. Thus, in total 29 tissues were available for analysis (Extended Data Table 1).

Prior differential expression analysis the expression data was limited to genes annotated as protein-coding or lncRNA genes in Gencode v19, further excluding all Y-chromosomal data as these genes are sex-specific by definition, resulting in total 27,334 genes. In addition, lowly-expressed genes, i.e. genes with median expression across samples ≤0.1 RPKM or expressed in fewer than 10 individuals at >1 counts per million, were excluded leaving between 13,067 and 16,157 genes per tissue for analysis (Extended Data Table 1).

Differential expression analysis between male and female samples was conducted using the voom-limma pipeline[7-9] available as an R package through Bioconductor (https://bioconductor.org/packages/release/bioc/html/limma.html): Gene-level read counts were first pre-processed using the voom function to stabilize the variance in the data and thereby to allow for the application of normal-based methods to RNA-seq data. Linear model was fitted using the log-transformed read counts and the precision weights from voom and differential expression analysis was conducted applying the lmFit and eBayes limma functions.

To remove technical and other unwanted variation, we adjusted the analyses for the following covariates: Age, three principal components inferred from genotype data using EIGENSTRAT[10], sample ischemic time, and surrogate variables[11,12] built using the sva R package[13] were used as continuous covariates and the cause of death classified into five categories based on the 4-point Hardy scale (samples where cause of death information was missing were categorized separately) as a categorical covariate. Surrogate variables were built separately for each tissue using age, principal components, ischemic time and cause of death as adjustment variables and sex as the variable of interest, and allowing sva to determine the number of latent factors that need to be estimated.

To control the false discovery rate (FDR), we used the qvalue R package to obtain q-values applying the adjustment separately for the differential expression results from each tissue. The null hypothesis was rejected for tests with q-values below 0.01.

Chromatin state analysis

To study the relationship between chromatin states and XCI, we used chromatin state calls from the Roadmap Epigenomics Consortium[14]. Specifically, we used the chromatin state annotations from the core 15-state model, publicly available at http://egg2.wustl.edu/roadmap/web_portal/chr_state_learning.html#core_15state. We followed our previously published method[15] to calculate the corrected percentage of each gene body assigned to each chromatin state. Briefly, for each gene and epigenome from the Roadmap Epigenomics Consortium, we calculated the percent of the gene body covered by each of the 15 chromatin states. Then, we corrected for covariates of sample type, sample state, and processing

center by calculating the deviance residual using logistic regression. After these pre-processing steps that were based on all 19,935 genes and 127 epigenomes, we filtered down to the 399 inactive and 86 escape genes on the X chromosome. Similarly, we filtered down to 38 female epigenomes and 51 male epigenomes, as described previously[15].

To compare the chromatin state profiles of the escape and inactive genes in female samples, we used the one-sided Wilcoxon rank sum test. Specifically, for each chromatin state, we averaged the chromatin state coverage across the 38 female samples for each gene. Then, we compared that average chromatin state coverage for all active genes to the average chromatin state coverage for all inactive genes. We performed both one-sided tests, to test for enrichment in escape genes, as well as enrichment in inactive genes.

Next, we performed simulations to account for any chromatin state biases, such as the fact that the escape and inactive genes are all from the X chromosome. Specifically, we generated 10,000 randomized simulations where we randomly shuffled the “escape” or “inactive” labels on the combined set of 485 genes, while retaining the sizes of each gene set. For each of these simulated “escape” and “inactive” gene sets, we calculated both Wilcoxon rank sum p-values as described above.

Then, we calculated a permutation “p-value” based on these 10,000 random simulations. Specifically, we calculated the percentile ranking of the p-value for our real data, compared to the simulated p-values. Formally:

$p_{perm} = (k+1)/(N+1)$

where k is number of simulations (out of 10,000) where $p_{sim} <= p_{real}$, and N is the number of simulations (10,000).

Finally, we used Bonferroni multiple hypothesis correction to correct for our 30 tests, one for each of 15 chromatin states, and both possible test directions. In other words, we considered a p_{perm} <0.00166 to be significant, as this new threshold was based on a cutoff 0.05/30.

Allele-specific expression

For ASE analysis the allele counts for biallelic heterozygous variants were retrieved from RNA-seq data using GATK ASEReadCounter (v.3.6)[16]. Heterozygous variants that passed VQSR filtering were first extracted for each sample from WES or WGS VCFs using GATK SelectVariants. The analysis was restricted to biallelic SNPs due to known issues in mapping bias in RNA-seq against indels[16]. Sample-specific VCFs and RNA-seq BAMs were inputted to ASEReadCounter requiring minimum base quality of 13 in the RNA-seq data (scRNA-seq samples, GTEX-UPIC) or requiring coverage in the RNA-seq data of each variant to be at least 10 reads, with a minimum base quality of 10 and counting only reads with unique mapping quality (MQ = 60) (clinical muscle samples).

For downstream processing of the scRNA-seq and GTEX-UPIC ASE data, we applied further filters to the data to focus on exonic variation only and to conservatively remove potentially spurious sites: we excluded all sites that 1) were not exonic, 2) had non-unique 25bp (for scRNA-seq samples) or 75bp (for GTEX-UPIC) mappability in the Human Female hg19 mappability track downloaded from http://www.broadinstitute.org/~anshul/projects/umap/, 3) had allele balance < 20, genotype quality < 40 or depth < 20 in the genotype data, 4) were not unique to protein-coding or lncRNA genes, with the exception of sites mapping to the *XIST* exon overlapping with *TSIX*, which were retained and considered unique to *XIST*, 5) had another SNP within 5bp potentially

indicating more complex pattern of variation at that site, or 6) in case of two SNPs within 25bp (or 75bp for GTEX-UPIC) of each other we only kept one to avoid one read spanning two ASE sites. For GTEX-UPIC we further limited the X-chromosomal ASE data in case of multiple ASE sites to only one site per gene, by selecting the site with coverage >7 reads in the largest number of tissues, to have equal representation from each gene for downstream analyses. Finally, after an initial analysis of the X-chromosomal ASE data we subjected 22 of the sites to manual investigation due to these providing potentially spurious results, i.e., results in conflict with previous surveys into XCI, which resulted in the exclusion of further five ASE sites.

Phasing scRNA-seq data

We assigned each cell to either of two cell populations distinguished by the parental X-chromosome designated for inactivation utilizing genotype phasing. For the YRI samples, where parental genotype data was available, the assignment to the two parental cell populations was unambiguous for all cells where X-chromosomal sites outside PAR1 or frequently biallelic sites were expressed. For 24A no parental genotype data was available, and hence we utilized the correlation structure of the expressed X-chromosomal alleles across the 948 cells to infer the two parental haplotypes utilizing the fact that in individual cells the expressed alleles at the chrX sites subject to full inactivation (i.e. the majority chrX ASE sites), are from the X chromosome active in each cell. Limitation of this approach is, however, that, unlike with the YRI trios, the parental origin of the haplotypes remains ambiguous. For this calculation we excluded all PAR1 sites and all additional sites that were frequently biallelic, to minimize the contribution of escape genes to the phase estimation.

Briefly, we 1) first estimated the relative phase of each ASE SNP pair by comparing the alleles detected at all cells where both sites were observed in the ASE analysis, which, given that not all SNP pairs could be compared, generated a sparse square matrix of concordances, and 2) then sequentially aggregated the haplotype phase information across the rows of the concordance matrix starting from the ASE SNP with the largest overall evidence for an accurate phase (estimated as the sum of absolute concordances across the ASE sites). 3) To estimate the accuracy of the above phasing approach (i.e. steps 1 and 2) we applied the approach 100 times using sets of 100 cells sampled from the full set of scRNA-seq ASE data. 4) To get the final estimated haplotype phase, we averaged the site-specific estimates from these 100 runs, which thus yielded a vector of probabilities of the reference allele at each site belonging to the same parental chromosome.

The alleles detected at each cell were then compared with this haplotype phase to determine which haplotype was active in each cell. The cell was assigned to the haplotype for which the sum of the number of reads for each allele weighted by the probability of the allele belonging to a given haplotype was greater.

After assigning each informative cell to either of the parental cell populations, the reference and alternate allele reads for each ASE site were mapped to active and inactive allele reads within each sample using the actual or inferred parental haplotypes. At sites where phase was unavailable (e.g. PAR1 sites for 24A that were not used for phasing or sites with ambiguous phase for YRI samples) the allele with the larger number of reads across cells, or in a case where cells from both cell populations express alleles at such site the allele combination (ref in population 1 + alt in population 2, or vice versa) with the larger number of reads, was assumed to be on the

active chromosome. As an exception, for 24A the expressed alleles at *XIST* were assigned to the inactive chromosome, in line with the pattern observed for YRI samples where parental genotype data confirms exclusive expression of *XIST* from Xi. The data was first combined per variant by taking the sum of active and inactive counts separately across cells, and further similarly combined per gene, if multiple SNPs per gene were available.

Manual curation of heterozygous variants from ASE analyses

Twenty-two heterozygous variants assessed in chrX ASE analysis were subjected to manual curation due to providing results in the XCI analysis that were in conflict with previous assignment of the underlying gene to be subject to full XCI. For each sample, BWA-aligned germline bams were viewed in IGV using WGS data if available, otherwise exome capture data were used. The locally realigned bams produced by HaplotypeCaller were also used, though the evidence from these was typically in agreement with the pileup from the BWA-aligned bams. The presence of a number of characteristics called into question the confidence of the variant read alignments and thus the variant itself. Allele balance that deviated significantly from 50:50 was considered suspect and often coincided with the existence of homology between the reference sequence in the region surrounding the variant and another area of the genome, as ascertained using the UCSC browser self-chain track and/or BLAT alignment of variant reads from within IGV. Other sequence-based annotations added to the VCF by HaplotypeCaller were also evaluated in the interests of examining other signatures of ambiguous mapping. Two variants were considered suspect based on low root-mean-squared mapping quality, or inferior mapping quality of reads supporting the alternate allele compared with those supporting the reference. In one case, a variant was also excluded from consideration because the base qualities of the alternate bases were significantly worse than those of the reference bases. The phasing of nearby variants was also considered. If phased variants occurred in the DNA sequencing data that were not assessed in the ASE analysis, those variants were considered suspect.

Further two SNPs that passed manual checks were excluded due to potentially spurious patterns in scRNA-seq as both lacked reference allele reads. For X:24578551 the expressed allele was assigned to the inactive paternal chromosome, and for X:48932564 all cells, independent of the parental X chromosome inactivated, expressed the alternative allele of the variant. While these can nevertheless represent real signals of incomplete inactivation such patterns can also be due to alignment or phasing artifacts and therefore were conservatively excluded.

Supplementary Tables

Supplementary Table 1. XCI status list compiled from previous studies. (provided separately)
Supplementary Table 2. Sex bias results for chrX from the GTEx analysis. (provided separately)
Supplementary Table 3. Nine genes that have not conclusively been described as escape genes in previous studies but follow a similar expression profile to escape genes in the GTEx sex bias analysis. (provided separately)
Supplementary Table 4: Variant QC for ASE. (provided separately)
Supplementary Table 5. X-chromosomal ASE results across 16 tissues in GTEX-UPIC. (provided separately)
Supplementary Table 6. Posterior probabilities for different ASE states for each X-chromosomal ASE site expressed in at least two tissues. (provided separately)
Supplementary Table 7. Association between posterior probabilities for different ASE states and XCI categories. Associations were assessed using generalized linear regression. (provided separately)
Supplementary Table 8. All scRNA-seq results for chrX. The data is summarized by gene for each sample. (provided separately)
Supplementary Table 9. Summary of observed XCI status in genes assessed in scRNA-seq samples. (provided separately)
Supplementary Table 10. Concordance of XCI status assignments from scRNA-seq with previous assignments. (provided separately)
Supplementary Table 11. New escape genes from scRNA-seq. Genes previously assigned as inactive that show significant Xi expression (see Methods for details) in at least one scRNA-seq sample are considered new candidate escape genes. (provided separately)
Supplementary Table 12. Xa and Xi expression between two X-chromosomal haplotypes in single cells. The difference in the level of Xi expression between the two haplotypes was compared in samples where both cell populations were present, i.e. XCI was not fully skewed, and at genes where both expression from Xi across all cells was significantly greater than baseline (binomial test P-value < 0.05, see Methods) and both haplotypes had coverage ≥ 8 reads. Significance between the expression from two haplotypes was assessed using 2-sample test for equality of proportions. (provided separately)
Supplementary Table 13. A summary of XCI analyses across the three data sets. Seven new escape gene candidates supported by evidence from at least two of the analyses are highlighted in orange. The potential characteristics supporting escape are 1) significant female bias in expression in GTEx population-level analysis, 2) posterior probability <0.5 for full inactivation across assessed tissues in chrX ASE data from GTEX-UPIC, and 3) significant Xi expression from at least one single cell sample. (provided separately)
Supplementary Table 14. List of antibodies used to perform single DC sorting in 96-well plates. (provided separately)

Supplementary Discussion

Factors potentially contributing to the heterogeneity in sex bias across tissues for escape genes

In the analysis of sex bias across GTEx tissues we identified a handful of established escape genes that showed uncharacteristically heterogeneous patterns of male-female expression differences, e.g. nonPAR genes *KAL1* and *ACE2* are both significantly male-biased in several tissues (Fig. 2a). Such a pattern can arise due to either subtler or more tissue-specific escape from XCI, yet we also find a few potential alternative explanations. Hormone-dependent gene regulation can hamper the detection of the expected female bias in escape gene expression. For instance, the predominant male-biased expression of *ACE2* is in line with the demonstrated higher *ACE2* activity in males partially driven by sex steroids[17]. We also note that a cluster of escape genes with less consistent sex bias profiles resides in the chromosomal region telomeric from the X-inactivation center, in the evolutionarily older region of the chromosome[18] (Fig. 2a and Extended Data Figure 5).

Magnitude of Xi expression along the chromosome

We find that level of expression from Xi varies with the chromosomal position of escape genes, as established previously[4,5]: mean Xi to Xa expression ratio peaks at PAR1 and nearby nonPAR regions, and then diminishes along the chromosome (Pearson r=-0.50, P=4.20E-03), consistent with the relatively subtle and heterogeneous sex bias in Xq compared to Xp (Fig. 2a). In nonPAR, the estimated level of Xi expression predicts the level of female bias in expression (Pearson r=0.78, P=2.62E-07), although, on average, in the population sample the magnitude of male-female expression difference at escape genes is slightly diluted from the allelic expression estimates for Xi expression (e.g. median in Xp 14% vs 21%).

Assessment of relative expression of the X and Y chromosome haplotypes of eleven PAR1 genes in skeletal muscle samples from eight males, leveraging a combination of RNA-seq and exome sequencing of trios (Methods), finds no evidence for systematic up or downregulation of Y chromosome expression (Extended Data Figure 8), thus indicating that the consistent male bias in PAR1 gene expression observed in the population-level analysis (Fig. 2a, female expression on average at 88% of male expression) is due to incomplete escape in PAR1 in females.

Implications of incomplete XCI for sex differences in health and disease

In our analyses of the GTEx data, we show that the incompleteness of XCI leads to expression differences between sexes for more than 60 genes, with sex bias often detected in multiple tissues and likely being present throughout life. As such, given that chances in gene regulation differences are key drivers of phenotypic differences, we suggest that incomplete XCI may well contribute to the widespread sex differences in health and disease.

Due to the unique inheritance pattern and the enrichment in haploinsufficient genes[19], there is an abundance of rare diseases attributable to X-chromosomal mutations. These predominantly affect males, yet the penetrance in females can be modulated by the skewness and incompleteness of

XCI[20,21]. Interestingly, several X-Y homologous genes have been implicated in severe diseases and syndromes, including established disease genes, such as *DDX3X* mutations in which cause intellectual disability[22] and *KDM6A* associated with Kabuki syndrome (OMIM 300128), both of which show considerable male bias in expression after accounting for the expression from the Y chromosome counterpart in the sex bias analysis.

Given the longstanding exclusion of the X chromosome from genome-wide association studies[23], the roles of X-chromosomal genes in complex traits, however, remain poorly understood. Despite this, X chromosome loci have been shown to contribute to several common phenotypes[24-26] and incompletely inactivated genes to contribute to sex differences[24-26].

Supplementary Note

Skewness of XCI in GTEx samples

Normal female tissue samples consist of two X-chromosomal cell populations, one where the paternal and one where the maternal X-chromosome is designated for inactivation. In the majority of cases the ratio of the two cell populations, i.e. skew in XCI, has been shown to mildly deviate from equal inactivation (50:50 ratio), yet considerable skews in XCI (>75% cells inactivate the same chromosome, “skewed XCI”) are relatively rare in population[27]. As such bulk tissue is rarely suitable for assessments of genic XCI status through allelic expression, as most heterozygous sites appear biallelically expressed due to the presence of the two active cell populations in the sample.

In order to characterize the patterns of XCI skew in GTEx female samples and to identify individuals with very skewed XCI (>95% of cells with the same inactive chromosome) therefore potentially informative for surveys into XCI, we utilized allelic expression in chrX measured via RNA-seq to get a measure for the ratio of the two cell populations in each female tissue sample. We hypothesized that allelic expression at a heterozygous site in an X-chromosomal gene subject to full X-inactivation is reflective of the ratio of the two parental cell populations in the tissue sample. The relative expression from each allele can, however, be modulated by regulatory variation. To account for the potential noise in allelic expression due to regulatory and other variation at individual genes, we used median allelic expression across all expressed (>=8 reads) nonPAR chrX sites as a proxy for the skew in XCI. Given that the majority of chrX genes are inactivated, median allelic expression should not be impacted by allelic expression from escape genes, however PAR1 data was excluded as these genes all escape from X-inactivation.

We used the multi-tissue GTEx RNA-seq data and and genotypes from Infinium ExomeChip to retrieve allelic read counts over heterozygous SNPs identified from genetic data. For genotyping, RNA-seq, and allele-specific expression details see[28]. All genotype and RNA-seq data utilized in this analysis is available in dbGap under accession phs000424.v3.p1. The X-chromosomal ASE data was summarized for each sample by taking the median allelic expression (i.e. |0.5 – (reference reads/total reads)|) for all nonPAR heterozygous SNPs covered by at least eight reads requiring at least three ASE sites qualifying these criteria for confident measure for XCI skew. Together XCI skew was estimated for 64 GTEx female donors and 42 unique tissue types.

The results grouped by tissue type and by individual are shown in Extended Data Figure 1. As established previously[5,29], most LCL samples demonstrate very skewed XCI due to their increased clonality, yet no other tissue type shows similar strong skewing, but rather most other samples fall within the normal range of skew (25-50% of cells with the same inactive chromosome), thus being unsuited for surveys of XCI via allelic expression. The per individual analysis, however, identified a highly unusual pattern in one of the female donors (ID: GTEX-UPIC), where the individual appears to have completely skewed XCI (i.e. median nonPAR allelic expression equal to zero) in all five tissue samples available in the GTEx V3 data. Further analyses showed that the very skewed XCI extends to all 16 tissues available in the later V6 data release and found no X-chromosomal abnormality in genotype data in this individual that could explain such a pattern. This unique sample was chosen for further analysis to interrogate sharing of XCI across tissues.

Supplementary References

1 Lee, M. N. *et al.* Common genetic variants modulate pathogen-sensing responses in human dendritic cells. *Science* **343**, 1246980, doi:10.1126/science.1246980 (2014).

2 Picelli, S. *et al.* Smart-seq2 for sensitive full-length transcriptome profiling in single cells. *Nat Methods* **10**, 1096-1098, doi:10.1038/nmeth.2639 (2013).

3 Trombetta, J. J. *et al.* Preparation of Single-Cell RNA-Seq Libraries for Next Generation Sequencing. *Curr Protoc Mol Biol* **107**, 4 22 21-17, doi:10.1002/0471142727.mb0422s107 (2014).

4 Carrel, L. & Willard, H. F. X-inactivation profile reveals extensive variability in X-linked gene expression in females. *Nature* **434**, 400-404, doi:10.1038/nature03479 (2005).

5 Cotton, A. M. *et al.* Analysis of expressed SNPs identifies variable extents of expression from the human inactive X chromosome. *Genome Biol* **14**, R122, doi:10.1186/gb-2013-14-11-r122 (2013).

6 Mele, M. *et al.* Human genomics. The human transcriptome across tissues and individuals. *Science* **348**, 660-665, doi:10.1126/science.aaa0355 (2015).

7 Law, C. W., Chen, Y., Shi, W. & Smyth, G. K. voom: Precision weights unlock linear model analysis tools for RNA-seq read counts. *Genome Biol* **15**, R29, doi:10.1186/gb-2014-15-2-r29 (2014).

8 Ritchie, M. E. *et al.* limma powers differential expression analyses for RNA-sequencing and microarray studies. *Nucleic Acids Res* **43**, e47, doi:10.1093/nar/gkv007 (2015).

9 Smyth, G. K. Linear models and empirical bayes methods for assessing differential expression in microarray experiments. *Stat Appl Genet Mol Biol* **3**, Article3, doi:10.2202/1544-6115.1027 (2004).

10 Price, A. L. *et al.* Principal components analysis corrects for stratification in genome-wide association studies. *Nat Genet* **38**, 904-909, doi:10.1038/ng1847 (2006).

11 Leek, J. T. & Storey, J. D. Capturing heterogeneity in gene expression studies by surrogate variable analysis. *PLoS Genet* **3**, 1724-1735, doi:10.1371/journal.pgen.0030161 (2007).

12 Leek, J. T. & Storey, J. D. A general framework for multiple testing dependence. *Proc Natl Acad Sci U S A* **105**, 18718-18723, doi:10.1073/pnas.0808709105 (2008).

13 Leek, J. T., Johnson, W. E., Parker, H. S., Jaffe, A. E. & Storey, J. D. The sva package for removing batch effects and other unwanted variation in high-throughput experiments. *Bioinformatics* **28**, 882-883, doi:10.1093/bioinformatics/bts034 (2012).

14 Roadmap Epigenomics, C. *et al.* Integrative analysis of 111 reference human epigenomes. *Nature* **518**, 317-330, doi:10.1038/nature14248 (2015).

15 Yen, A. & Kellis, M. Systematic chromatin state comparison of epigenomes associated with diverse properties including sex and tissue type. *Nat Commun* **6**, 7973, doi:10.1038/ncomms8973 (2015).

16 Castel, S. E., Levy-Moonshine, A., Mohammadi, P., Banks, E. & Lappalainen, T. Tools and best practices for data processing in allelic expression analysis. *Genome Biol* **16**, 195, doi:10.1186/s13059-015-0762-6 (2015).

17 Liu, J. *et al.* Sex differences in renal angiotensin converting enzyme 2 (ACE2) activity are 17beta-oestradiol-dependent and sex chromosome-independent. *Biol Sex Differ* **1**, 6, doi:10.1186/2042-6410-1-6 (2010).

18 Ross, M. T. *et al.* The DNA sequence of the human X chromosome. *Nature* **434**, 325-337, doi:10.1038/nature03440 (2005).

19 Lek, M. *et al.* Analysis of protein-coding genetic variation in 60,706 humans. *Nature* **536**, 285-291, doi:10.1038/nature19057 (2016).

20 Deng, X., Berletch, J. B., Nguyen, D. K. & Disteche, C. M. X chromosome regulation: diverse patterns in development, tissues and disease. *Nat Rev Genet* **15**, 367-378, doi:10.1038/nrg3687 (2014).

21 Dobyns, W. B. *et al.* Inheritance of most X-linked traits is not dominant or recessive, just X-linked. *Am J Med Genet A* **129A**, 136-143, doi:10.1002/ajmg.a.30123 (2004).

22 Snijders Blok, L. *et al.* Mutations in DDX3X Are a Common Cause of Unexplained Intellectual Disability with Gender-Specific Effects on Wnt Signaling. *Am J Hum Genet* **97**, 343-352, doi:10.1016/j.ajhg.2015.07.004 (2015).

23 Wise, A. L., Gyi, L. & Manolio, T. A. eXclusion: toward integrating the X chromosome in genome-wide association analyses. *Am J Hum Genet* **92**, 643-647, doi:10.1016/j.ajhg.2013.03.017 (2013).

24 Chang, D. *et al.* Accounting for eXentricities: analysis of the X chromosome in GWAS reveals X-linked genes implicated in autoimmune diseases. *PLoS One* **9**, e113684, doi:10.1371/journal.pone.0113684 (2014).

25 Kukurba, K. R. *et al.* Impact of the X Chromosome and sex on regulatory variation. *Genome Res* **26**, 768-777, doi:10.1101/gr.197897.115 (2016).

26 Tukiainen, T. *et al.* Chromosome X-wide association study identifies Loci for fasting insulin and height and evidence for incomplete dosage compensation. *PLoS Genet* **10**, e1004127, doi:10.1371/journal.pgen.1004127 (2014).

27 Amos-Landgraf, J. M. *et al.* X chromosome-inactivation patterns of 1,005 phenotypically unaffected females. *Am J Hum Genet* **79**, 493-499, doi:10.1086/507565 (2006).

28 Consortium, G. T. Human genomics. The Genotype-Tissue Expression (GTEx) pilot analysis: multitissue gene regulation in humans. *Science* **348**, 648-660, doi:10.1126/science.1262110 (2015).

29 Baran, Y. *et al.* The landscape of genomic imprinting across diverse adult human tissues. *Genome Res* **25**, 927-936, doi:10.1101/gr.192278.115 (2015).
